# Improving the Efficacy of Tumor Radiosensitization Through Combined Molecular Targeting

**DOI:** 10.3389/fonc.2020.01260

**Published:** 2020-08-04

**Authors:** Katharina Hintelmann, Malte Kriegs, Kai Rothkamm, Thorsten Rieckmann

**Affiliations:** ^1^Laboratory of Radiobiology & Experimental Radiation Oncology, University Medical Center Hamburg Eppendorf, Hamburg, Germany; ^2^Department of Otolaryngology and Head and Neck Surgery, University Medical Center Hamburg Eppendorf, Hamburg, Germany

**Keywords:** radiotherapy, radioresistance, radiosensitization, combined molecular targeting, dual inhibition

## Abstract

Chemoradiation, either alone or in combination with surgery or induction chemotherapy, is the current standard of care for most locally advanced solid tumors. Though chemoradiation is usually performed at the maximum tolerated doses of both chemotherapy and radiation, current cure rates are not satisfactory for many tumor entities, since tumor heterogeneity and plasticity result in chemo- and radioresistance. Advances in the understanding of tumor biology, a rapidly growing number of molecular targeting agents and novel technologies enabling the in-depth characterization of individual tumors, have fuelled the hope of entering an era of precision oncology, where each tumor will be treated according to its individual characteristics and weaknesses. At present though, molecular targeting approaches in combination with radiotherapy or chemoradiation have not yet proven to be beneficial over standard chemoradiation treatment in the clinical setting. A promising approach to improve efficacy is the combined usage of two targeting agents in order to inhibit backup pathways or achieve a more complete pathway inhibition. Here we review preclinical attempts to utilize such dual targeting strategies for future tumor radiosensitization.

## Introduction

Chemoradiation is a current standard of care for the curative treatment of most locally advanced solid malignancies. Both modalities are generally administered at the maximum-tolerated doses to achieve best possible cure rates, which for many entities such as lung, brain, colorectal, bladder, or human Papillomavirus (HPV)-negative head and neck cancer, are still far from satisfactory. Due to the intense treatment regimes a considerable fraction of patients suffer from severe acute as well as late and partly irreversible side effects that can seriously impact quality of life. For example in head and neck squamous cell carcinoma (HNSCC) the addition of platin-based chemotherapy to radiotherapy increases 5-year overall survival by about 10% (1, 2) at the cost of increases in the rate of severe adverse events, such as grade 3 mucositis, anemia and nephro- and ototoxicity, which can result in lifetime renal insufficiency and hearing loss (3).

Combining radiotherapy with molecular targeting agents may offer an alternative to chemoradiation with potentially less severe side effects, provided the tumor cells are more dependent on the specific target than normal tissue. To be effective, the targeting agent needs to be directly toxic for the tumor and/or has to induce a meaningful radiosensitization. Despite a plethora of promising preclinical data, the results achieved in the clinic are so far exceedingly disappointing. The only currently approved molecular targeting agent for the combination with radiotherapy is the anti-epidermal growth factor receptor (EGFR)-antibody cetuximab in HNSCC. The combination was approved on the basis of the IMC 9815 phase III clinical trial, which demonstrated superiority over radiation alone in a range similar to the addition of cisplatin to radiotherapy (4). However, after a considerable number of subsequent publications it has to be seriously called into question whether the addition of cetuximab to radiotherapy is a viable alternative for cisplatin (5–7) and cetuximab also failed to enhance survival when added to chemoradiation (8). Recently, cetuximab-radiation was directly shown to be inferior to cisplatin-based chemoradiation in HPV-positive oropharyngeal cancer in two prospective phase III trials (9, 10) although this entity had shown the greatest benefit from cetuximab in the IMC 9815 trial (11).

A general limitation for the effective use of molecular targeted agents is the current lack of biomarkers that could predict a possible oncogenic addiction to a given druggable target or a possible role of the target in radiation resistance. Also in the case of cetuximab in HNSCC, no predictive biomarker has been established. In order to fully exploit the potential of precision medicine, such biomarkers are mandatory to select the best agents for a given tumor. Sequencing individual tumors for druggable driver mutations is one way forward. However, to what extent the targeting of such potential oncogenic driver proteins will also result in an enhanced sensitivity toward radiotherapy is currently unknown.

Another important concern is therapy resistance due to backup pathways or incomplete inhibition. In such cases, combined molecular targeting approaches may be an effective way to increase efficacy. Combined targeting often follows three main strategies: (1) blocking of potential alternative pathways, (2) dual targeting of the same pathway to achieve a more complete inhibition or (3) targeting of two distinct pathways whose dual inhibition will result in synthetic lethality or synergistic radiosensitization (12).

Here we review preclinical attempts to utilize such dual targeting strategies for future tumor radiosensitization.

## Methods

**Figure 1:**
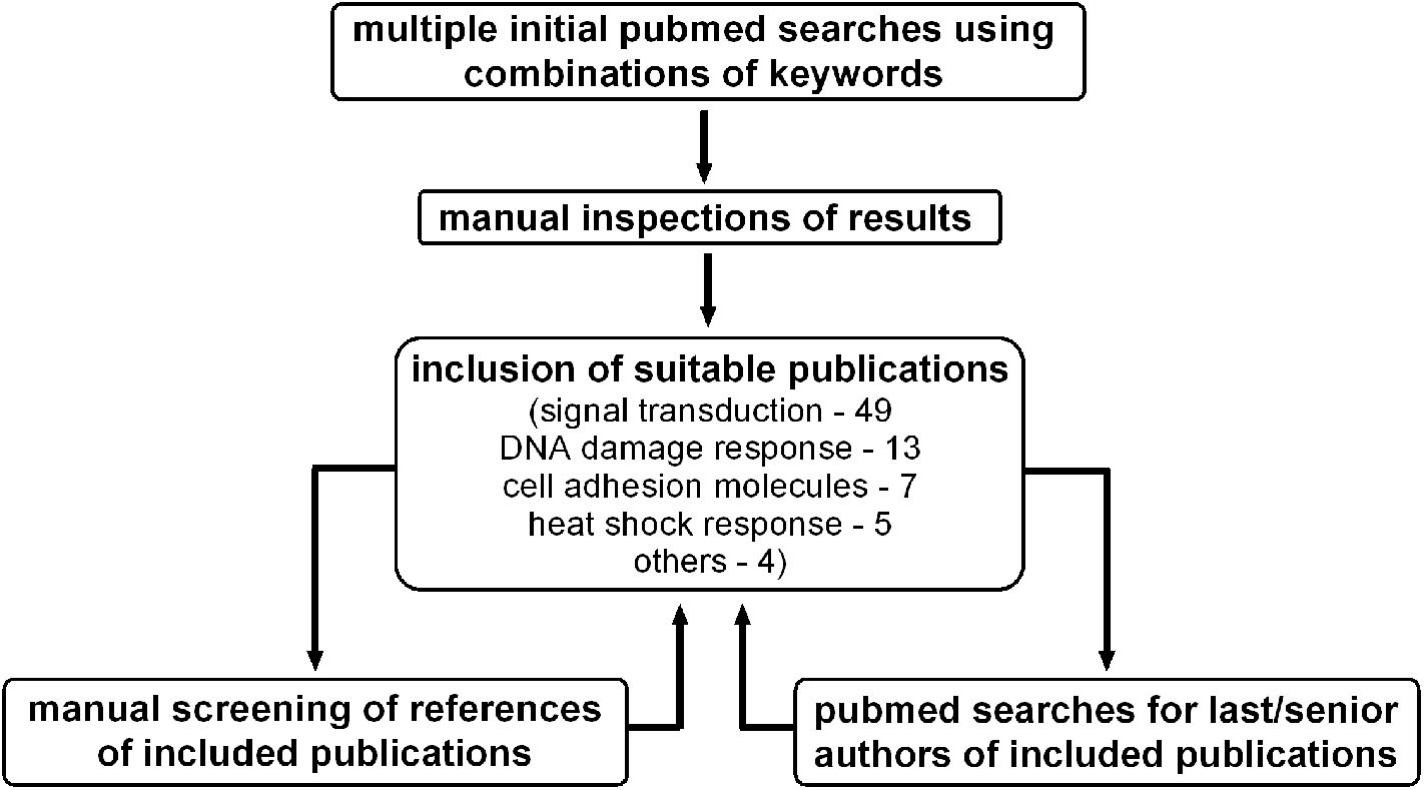
Screening process for preclinical publications utilizing combined molecular targeting approaches for tumor radiosensitization.

A PubMed search based on the key words “agent^*^, radiosensiti^*^, radiotherapy, molecular targeted therapy, combined molecular targeting” was conducted and the results were screened for use of combined molecular targeting for radiosensitization in the preclinical setting. In addition, because titles and abstracts do not follow any regular pattern, references from identified articles were further screened for suitable publications and PubMed was additionally screened for publications from the last/senior authors of identified articles (Figure 1).

Publications dealing with immunotherapy, e.g., using immune checkpoint inhibitors were not included, since they do not represent radiosensitization in the narrow sense. Publications of combined usage of molecular targeting and chemotherapy to achieve radiosensitization were also not included. Further, it was not always possible to discriminate between the intentional combined inhibition of two defined molecular targets and the less well-defined usage of somewhat unspecific agents with two or more targets. The latter were considered when reflecting the basic idea of the combined targeting approaches for radiosensitization, i.e., the intended selection of two targets whose inhibition should achieve at least additive or even synergistic effects.

Regarding clinical trials with published results, we performed a PubMed search with the respective targeting agents found in preclinical studies plus the terms “radiation” or “radiotherapy.” Since the focus of this review is on preclinical approaches, we only present a selection of the most important clinical trials.

## Results

The vast majority of publications reporting experimental dual targeting approaches in combination with ionizing radiation fall into four categories: (1) growth factor receptor signaling, (2) DNA damage response and cell cycle checkpoints, (3) cell adhesion molecules, and (4) the heat shock response. From these categories targeting growth factor receptor signaling currently represents the by far most extensively studied dual targeting approach. In some of the identified papers inhibitors belonging to two of these categories were combined. These papers will only be presented in one section. Studies using a single substance with dual specificity were considered when its use was based on a rational selection of targets whose inhibition should achieve at least an additive or a synergistic effect.

### Targeting Growth Factor Receptor Signaling

**Figure 2:**
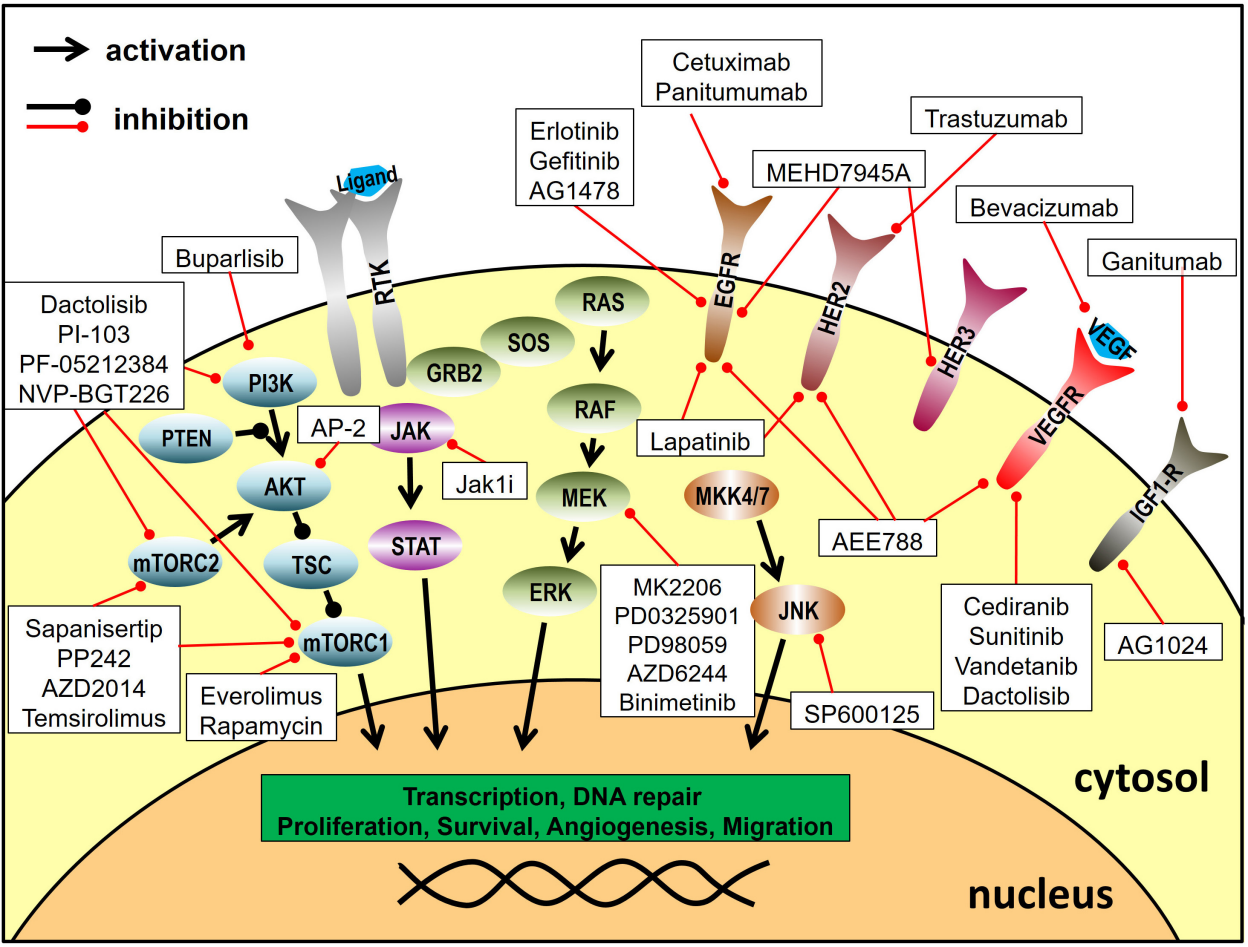
Targeting of signal transduction pathways. Depicted are the inhibitors utilized for combined molecular targeting approaches for tumor radiosensitization and their respective target proteins. Reported inhibitor combinations for radiosensitization are described in the text and are listed in Table 1. RTK, receptor tyrosine kinase.

The most frequently used approach of radiosensitization through dual molecular targeting is the inhibition of growth factor receptor tyrosine kinases and their related signaling pathways. Growth factor receptor signaling can contribute to radioresistance, because it stimulates proliferation, inhibits apoptosis and has been described to increase the repair of radiation-induced DNA-damage, which makes it an attractive molecular target for radiosensitization (13, 14). Combined targeting approaches were further fuelled by the approval of the anti-EGFR monoclonal antibody (mAb) cetuximab in the curative treatment of HNSCC and by the desire to increase efficacy and repress by-pass signaling and resistance, which pose a potential risk to all signaling inhibition approaches (15). Figure 2 provides an overview of the inhibited signaling pathways and proteins described in this section.

**Table 1:** Combined targeting of growth factor receptor signaling.

**Targets**	**Inhibitor(s)**	**Entity**	**References**
EGFR/HER2	Gefitinib^**^,^***^ Trastuzumab^**^,^***^	Vulvar squamous cell carcinoma	(20)
EGFR/HER2	Lapatinib^**^,^***^	Breast cancer	(21, 22, 24)
		Breast cancer (HER2+)	(25)
		K- pancreatic cancer (K-ras wt)	(23)
		NF2 associated peripheral nerve sheath tumor	(26)
		Bladder cancer	(27)
EGFR/HER3	MEHD7945A^*^	NSCLC, HNSCC	(28, 29)
EGFR/IGF-1R	Panitumumab^**^,^***^ Ganitumab^*^	HNSCC	(31)
	Erlotinib^**^,^***^ AG1024^exp^	Prostate cancer	(32)
	AG1478^exp^ AG1024^exp^	Breast cancer	(33)
EGFR/JAK/ STAT-3	Cetuximab^**^,^***^ JAK1i^exp^	HNSCC	(37)
EGFR/JNK2/ JIP-4	Cetuximab^**^,^***^ SP600125^exp^	HNSCC/VSCC	(38)
EGFR/mTOR	Erlotinib^**^,^***^ Everolimus^**^,^***^	NSCLC?	(39)
EGFR/VEGFR	Gefitinib^**^,^***^ ZD6126^*^^(*sus*)^	HNSCC	(48)
	Gefitinib^**^,^***^ AZD2171 (Cediranib)^**^	HNSCC	(44)
	Erlotinib^**^,^***^ Bevacizumab^**^,^***^	HNSCC	(45)
	Cetuximab^**^,^***^ Sunitinib^**^,^***^	HNSCC	(46)
	Cetuximab^**^,^***^ Bevacizumab^**^,^***^ Temsirolimus^**^,^***^	HNSCC	(47)
	Vandetanib^**^,^***^	NSCLC	(49)
	Vandetanib^**^,^***^	GBM	(51)
EGFR/VEGFR/ HER2	AEE788^*^^(disc)^	Mammary carcinoma (murine)	(50)
mTOR1C/mTOR2C	Sapanisertib^*^	Pontine Glioma	(56)
		Breast Cancer	(57)
	PP242^exp^	Breast Cancer	(58)
	AZD2014 (Vistusertib)^*^	Glioblastoma	(59)
PI3K/mTOR[(ATM/DNAPK_CS_)]	Dactolisib^*^	Oral SCC	(60)
		HNSCC	(70)
		Glioblastoma	(61, 62, 66)
		NSCLC	(64)
		Colorectal cancer	(65)
		Prostate cancer	(67, 68)
		Fibrosarcoma, HNSCC	(75)
	Dactolisib^*^ NVP-BGT226^^*^(*disc*)^	HNSCC, bladder cancer, endothelial cells	(76)
	Dactolisib^*^ PI-103^exp^	Prostate cancer	(69)
PI3K/mTOR	PI-103^exp^	Colon cancer	(72)
	PF-05212384 (Gedatolisib)^*^	HNSCC	(73)
	PF-04691502^*^^(*disc*)^	HNSCC	(74)
PI3K/mTOR/PARP	PI-103^exp^ Olaparib^**^,^***^	TNBC	(71)
mTOR/Akt	Rapamycin^**^,^***^ MK2206^*^	NSCLC, breast cancer	(77)
MEK/Akt	PD0325901^*^ API-2 (=Triciribine)^*^	Pancreatic cancer (K-ras mut.)	(78)
PI3K/mTOR/MEK	PI-103^exp^ PD98059^exp^	K-ras mut. NSCLC	(79)
	Dactolisib^*^ AZD6244 (Selumentinib)^**^,^***^	Lung cancer, Glioblastoma	(80)
PI3K/MEK	Buparlisib^**^ Binimetinib^**^,^***^	HNSCC	(81)

* *Tested in clinical trials*.

** *Tested in clinical trials in combination with radiotherapy*.

*** *Approved (any clinical setting)*.

*exp, experimental; disc, discontinued; sus, suspended*.

#### The HER Family

The HER sub-family of receptor tyrosine kinases (RTK) includes the members EGFR (also termed HER1 or ErbB1), Her2 (ErbB2), HER3 (ErbB3), and HER4 (ErbB4). These transmembrane receptors are located at the cell surface, harbor an intrinsic protein kinase domain and regulate proliferation, migration, cell fate determination and apoptosis via diverse downstream signaling pathways such as MAPK and AKT signaling (16). EGFR is expressed in normal epithelial cells of the skin, hair follicles or the gastro intestinal tract, but it is also detected in many tumor entities. Furthermore, EGFR gene amplifications or mutations are found in e.g., HNSCC, lung cancer or glioblastoma (GBM), driving carcinogenesis and tumor progression (17, 18). Consequently, targeting EGFR with mAbs or tyrosine kinase inhibitors (TKI) has been established in cancer therapy in e.g. NSCLC, colorectal cancer, head and neck cancer, or pancreatic cancer but therapy resistance occurs frequently and compromises outcome (19). Usually, ligand-binding leads to ErbB receptor homodimerization but can also result in the formation of heterodimers consisting of different sub-family members. Due to these interactions and possible functional redundancies co-targeting of different sub-family members has been investigated in several pre-clinical studies.

##### HER/HER targeting

Combined inhibition of different members of the HER sub-family indeed showed promising results in terms of radiosensitization. For example in first studies Fukutome et al. combined the EGFR inhibitor gefitinib (TKI) and the anti-HER2 antibody trastuzumab. Both inhibitors induced radiosensitization on their own and their combination resulted in a synergistic sensitization in vulvar squamous cell carcinoma cells expressing EGFR and HER2 (20).

Also EGFR/HER-2 inhibition by the dual inhibitor lapatinib resulted in enhanced radiosensitivity in cancer cells of various entities, such as bladder cancer, peripheral nerve sheath tumors, pancreatic or breast cancer. This sensitization was shown to be partly dependent on the expression of the specific targets (HER2, EGFR) and to be inhibited through the constitutive activation of downstream signaling factors, such as Ras & Raf mutations (21–27).

To inhibit EGFR and HER3 Huang et al. used the dual inhibitor MEHD7945A. They demonstrated that MEHD7945A inhibits growth in cetuximab (EGFR mAb) and erlotinib (EGFR TKI) resistant cells with a significant PI3K and MAPK pathway inhibition. In a xenograft model, MEHD7945A reduced the growth of tumors resistant to mono-EGFR-targeting, and, in contrast to cetuximab, the combination with radiation resulted in a more pronounced growth inhibition than either modality alone. EGFR and HER3 are both activated upon radiation and the blockade of one receptor may be compensated by the other. Treatment with MEHD7945A but not with cetuximab reduced survival signaling and DNA repair (28). The same group could substantiate the evidence for a radiosensitizing effect of MEHD7945A using human lung and head and neck cancer cells as well as xenografts further supporting the clinical implementation of this EGFR/HER3 combined targeting approach (29).

##### HER/IGF-1R targeting

In addition to the formation of heterodimers within the HER-family there is also a cross talk between EGFR and other receptor tyrosine kinases such as the insulin like growth factor 1 receptor (IGF-1R), which is also involved in tumor development and progression (30). In this context Matsumoto et al. compared individual and dual targeting of EGFR and IGF-1R in an HNSCC xenograft model using the mAbs ganitumab (anti-IGF-1R) and panitumumab (anti-EGFR). They observed the strongest growth arrest and significantly fewer recurrences upon combined inhibition plus radiation (31). Wang et al. also showed a radiosensitizing effect of combined inhibition of EGFR through erlotinib and the IGF-1R inhibitor AG1024 in prostate cancer cells, suggesting a suppression of homologous recombination repair as a possible underlying mechanism (32). Using two breast cancer cell lines with similarly high expression of IGF-1R but differential expression of EGFR, Li et al. observed radiosensitization through IGF-1R-inhibition (AG1024) in both strains. The EGFR inhibitor AG1478, however, only radiosensitized the cell line with high EGFR-expression both alone and when added to IGF-1R-inhibition. Radiosensitization through combined targeting was further validated in a xenograft model (33).

##### HER/downstream targeting

The HER receptors transduce their signals through several downstream pathways including the Ras-Raf-MAPK, the PI3K-Akt and the JAK/STAT pathway (19, 34). Alterations within these pathways might affect the efficacy of HER inhibition as demonstrated by the importance of the Ras mutation status in colorectal cancer where patients carrying such mutations do not benefit from cetuximab treatment (35, 36). Therefore, another strategy to increase efficacy is to combine the inhibition of the receptors and relevant downstream targets.

In this context Bonner et al. assessed the effect of combined treatment of head and neck cancer cells with cetuximab and the JAK inhibitor JAK1i. STAT3 is a downstream protein activated by JAK (among others) protecting cells from apoptosis. The authors observed enhanced anti-proliferative and apoptotic effects upon dual inhibition plus radiation. Dual inhibition was accompanied by a more complete inhibition of STAT3-phosphorylation and, in contrast to single inhibition, resulted in radiosensitization in colony formation assays (37).

Eke et al. identified the activation of c-Jun N-terminal kinase 2 (JNK2) via the scaffold protein JNK-interacting protein 4 (JIP-4) as a possible signaling bypass after EGFR targeting. The authors knocked down JIP4 or JNK2 via siRNA and used the JNK2 inhibitor SP600125 in addition to cetuximab treatment and achieved enhanced tumor cell radiosensitization in an additive manner as compared to single inhibition (38).

Activation of the PI3K/Akt/mTOR pathway was demonstrated by Zhuang et al. in lung adenocarcinoma cells as another resistance mechanism against EGFR targeting. They could demonstrate that mTOR inhibition with everolimus enhanced radiation sensitivity when added to erlotinib *in vitro* and in a xenograft model (39).

##### HER/VEGF(R) targeting

The family of vascular endothelial growth factors (VEGFs) and their specific receptors (VEGFRs) are frequently targeted in cancer therapy, e.g., in lung, breast, kidney, ovarian and cervix cancer. A fundamental difference in this therapeutic strategy is that, although the inhibition of tumor cell signaling is also of relevance, the main target of VEGF(R)-inhibition is tumor angiogenesis. VEGFs and VEGFRs are critical factors in the formation and maintenance of new vasculature in both normal tissues and solid tumors (40, 41). Their inhibition can indeed follow two contrary intentions: (1) a complete inhibition resulting in depletion of tumor nutrient and oxygen supply, or (2) a partial inhibition that results in normalization of tumor vasculature, enhances oxygenation and decreases hypoxia-based radiation resistance. Some rationales have been described for combining VEGF and EGFR inhibition. Amongst others, EGFR is also involved in angiogenesis and it has been described that EGFR inhibitor resistance may be associated with VEGF up-regulation and angiogenesis (42, 43).

In this context Bozec et al. demonstrated promising results using the VEGFR inhibitor cediranib (AZD2171) (targeting VEGFR1/2/3) concurrent with the EGFR inhibitor gefitinib and radiotherapy in a VEGF secreting HNSCC xenograft model. Combined treatment plus radiation clearly inhibited tumor growth more effectively than dual or single inhibition or radiotherapy alone. Dual inhibition was associated with decreased vessel density and dual inhibition plus irradiation showed the highest decrease in proliferation as assessed by Ki67 staining (44). The group could confirm the radiosensitizing effects in further studies when treating the same VEGF-secreting HNSCC model as orthotopic xenografts using alternative, but functionally equivalent agents, namely the anti-VEGF monoclonal antibody bevacizumab combined with the EGFR TKI erlotinib or using the combination of the VEGFR TKI sunitinib and the EGFR mAb cetuximab (45, 46). Due to an observed tumor re-growth associated with AKT/mTOR signaling activation, they further investigated the triple-targeting approach of cetuximab, bevacizumab, and the mTOR inhibitor temsirolimus in combination with irradiation. Adding the third inhibitor they indeed achieved the most sustained growth inhibition (47). In previous studies the same group had combined ZD6126, an antivascular tubulin-binding agent, with the EGFR TKI inhibitor gefitinib and irradiation. In contrast to the results described above, and although the combined targeting was moderately more effective than single targeting, the addition of radiation to dual targeting did not result in a further reduction of tumor growth (48).

Radiosensitiziation could also be induced in a lung cancer model by vandetanib, an inhibitor of VEGFR2 and EGFR but also of RET and other receptors. In human lung adenocarcinoma vandetanib treatment added to radiotherapy resulted in a dose enhancement ratio of 1.32 and markedly inhibited sublethal damage repair as assessed by a split dose recovery assay. *In vivo* the combination with irradiation showed enhanced tumor growth inhibition as compared to single treatment (49). Oehler et al. tested the effect of AEE788, an inhibitor of EGFR, HER2 and VEGFR, plus irradiation in a spontaneously growing murine mammary carcinoma model and in tumor allografts derived from murine mammary carcinoma cells. AEE788 alone as well as in combination with radiation improved tumor oxygenation in both models and the combined treatment resulted in an at least additive tumor response. Using specific inhibitors, the improvement of oxygenation could be assigned to the EGFR/HER2 inhibition (50).

In U87 GBM cell lines with or without ectopic EGFR expression vandetanib as well as cediranib failed to induce radiosensitization in clonogenic assays indicating no effect on DNA repair. In the respective xenograft models only the combination of vandetanib plus irradiation reduced tumor growth more strongly than irradiation alone, and only in the EGFR expressing substrain. In line with reduced tumor growth in this model system, vandetanib but not cediranib suppressed the expression levels of pAkt, survivin, and Ki67 as well as VEGF secretion (51).

#### The PI3K-AKT-mTOR Pathway

The stimulation of various growth factor receptors leads to the activation of the PI3K-AKT-mTOR signaling pathway, which can cause resistance to apoptosis and radiation. Elevated activity of the PI3K-AKT-mTOR pathway is observed in a broad range of tumor entities and associated with poor outcome, which makes this pathway a promising target for inhibitory strategies (52–55).

##### mTORC1/mTORC2

Inhibition of the PI3K-AKT-mTOR pathway is usually achieved by mTOR inhibitors, such as rapamycin or everolimus. However, these inhibitors block the mTOR Complex1 (mTORC1), which often results in the up-regulation of the mTOR Complex 2. Therefore, combined inhibition of mTOR Complex 1 and 2 has been studied using dual inhibitors. Sapanisertib is an ATP-competitive mTORC1 and mTORC2 inhibitor. Miyahara et al. demonstrated an enhanced inhibition of proliferation and induction of apoptosis when combining the dual inhibitor and radiation in diffuse intrinsic pontine glioma cells (56). Liu et al. also showed a radiosensitizing effect of sapanisertib in breast cancer cells, which was associated with G2/M cell-cycle arrest and an inhibition of DNA double-strand break (DSB) repair (57).

Hayman et al. compared the radiosensitization through the mTORC1-inhibitor rapamycin and the dual mTORC1/mTORC2 inhibitor PP242 in breast cancer cell lines and only observed a radiosensitizing effect using the dual inhibitor. As a normal tissue cell control, lung fibroblasts were not radiosensitized through PP242 treatment. *In vivo* PP242 alone had no impact on tumor growth but enhanced the radiation-induced growth reduction (58). The same group also tested an alternative mTORC1/mTORC2 inhibitor, AZD2014, which induced radiosensitization in glioblastoma stem-like cells *in vitro* and *in vivo*. A delay in the dispersal of radiation-induced γH2AX foci suggests that this effect involves the inhibition of DNA repair (59).

##### PI3K/mTOR targeting

In addition to dual targeting of mTORC1 and mTORC2 the combination of inhibitors targeting different players of the PI3K-AKT-mTOR pathway are under highly intensive investigation. In this context Yu et al. examined the effect of the dual PI3K/mTOR inhibitor dactolisib (NVP-BEZ235) in patient-derived and in radioresistant oral squamous cell carcinoma cells *in vitro* and in an *in vivo* tumor model. They observed radiosensitization *in vitro*, associated with G1 phase arrest by the downregulation of cyclin D1/CDK4 complex as a consequence of the PI3K/mTOR signaling inhibition. Tumor shrinkage was more pronounced upon the combination of dactolisib and radiation as compared to radiation alone (60). Dactolisib was further shown to reduce the activity of the central DNA repair factors DNA-PKcs and ATM and, as a consequence, to efficiently block the repair of IR-induced DSBs. Consequently, an effective radiosensitization could be demonstrated in glioblastoma cells *in vitro* and *in vivo* (61, 62).

Aberrant activation of the PI3K/AKT/mTOR pathway by Ras mutations is an important factor in Ras-driven tumorigenesis (63). Using dactolisib, Konstantinidou et al. could demonstrate a more effective radiosensitization of K-ras mutant NSCLC cells as compared to the single inhibition of PI3K (LY294002) or mTOR (rapamycin). *In vivo* dactolisib alone had little effect on tumor growth but profoundly enhanced the effect of irradiation (64). Substantiating this data, Chen et al. also targeted PI3K and mTOR with dactolisib using K-ras mutant and wild type colorectal cancer cells. Dactolisib had a radiosensitizing effect in both cases. They further demonstrated the same effect in a xenograft tumor model and suggested inefficient DNA repair, possibly due to impaired activation of ATM and DNAPKcs upon dactolisib treatment (65). In glioblastoma cell lines the radiosensitizing effect of dactolisib was shown to be dependent on the scheduling of drug and radiation. A 24 h preincubation period and wash out of the drug right before irradiation and seeding failed to sensitize the cells, while the addition of the drug shortly (1 h) before radiation with subsequent incubation for 24 h before seeding was highly effective. In line with the colony formation data, only the latter schedule showed reduced levels of P-AKT and P-mTOR without and 30 min after irradiation (66). Potiron et al. used dactolisib *in vitro* and *in vivo* in prostate cancer cell lines under normoxic and hypoxic conditions. They found a radiosensitizing effect in all cases and observed a reduction in DSB repair associated with an enhanced G2 cell cycle arrest (67). Comparable results in prostate cancer cell lines were reported in two further studies, supporting the theory of an impaired DNA repair capacity (68, 69). Schötz et al. observed radiosensitization in HNSCC cell lines, regardless of HPV-status. A DNA-repair defect was more apparent in the G1 than G2 phase and reporter gene assays pointed toward inhibition of non-homologous endjoining (NHEJ), but not homologous recombination (HR) (70). Chang et al. also tested an alternative dual PI3K/mTOR inhibitor, PI-103, which caused radiosensitization comparable to dactolisib. They suggested a novel mechanism of radiosensitization based on a reduced expression of NHEJ (Ku70/80), as well as HR (BRCA1/2, Rad51) factors upon PI3K/mTOR inhibition and radiation (69). Along the same line Jang et al. reported a severely reduced BRCA1 expression upon PI-103 treatment and a radiosensitzation that could be further augmented by PARP-inhibition through olaparib. PI-103 failed to induce radiosensitization after a preceeding siRNA-mediated knockdown of BRCA1 suggesting that BRCA1/HR is the most relevant target in this regard (71). PI-103 was also shown to radiosensitize colon cancer cells with activated AKT through inhibition of DSB repair (72).

Leiker et al. analyzed a third ATP-competitive dual PI3K-mTOR inhibitor, PF-05212384. Using HNSCC cells they demonstrated delayed γH2AX foci resolution and a significant radiosensitization *in vivo* and *in vitro*. Since the effect was more pronounced in tumor cells compared to normal fibroblasts the results indicate some degree of tumor specificity (73). A differential response in two HNSCC cell lines toward the PI3K-mTOR inhibitor, PF-04691502 was described by Tonlaar et al. While one strain was sensitized, the other failed to respond, in line with an increased constitutive activity of PI3K, AKT, and mTOR and an inability to inhibit key phosphorylation events upon treatment (74).

Following a concept of PI3K/mTOR inhibition different from the ones described above, Fokas et al. used dactolisib as an alternative to VEGFR-inhibition in order to induce vascular normalization and improved oxygen supply. *In vivo* they observed a reduction in tumor hypoxia and an increase in perfusion. Using different schedules of drug treatment and irradiation that did or did not provide adequate time for vascular remodeling, they observed differences in tumor growth delay and concluded that dactolisib is capable of both, radiosensitization through vasculature normalization and in a direct manner (75). The same group further characterized this direct effect in a panel of different tumor and endothelial cells using dactolisib and another dual PI3K/mTOR inhibitor, NVP-BGT226. They observed PI3K pathway inhibition and enhanced residual γH2AX foci and G2-arrest after irradiation. Human endothelial and dermal microvascular cells were also sensitized, which suggests possible effects on tumor vasculature but may also indicate sensitization of normal tissue cells, which urges caution, when progressing to clinical trials (76).

##### AKT/mTOR

Another possibility for highly effective targeting of the PI3K-AKT-mTOR pathway is the combined inhibition of AKT and mTOR. Upon treatment with the mTOR-inhibitor rapamycin, Holler et al. observed an activation of Akt in cell lines that showed no or little radiosensitization. Since this activation was not present in responsive cells, they combined rapamycin with the Akt-inhibitor MK2206 and observed radiosensitization and an enhanced number of residual DSBs (77).

##### Combined inhibition of the PI3K/AKT/mTOR and Ras/Raf/Mek/MAPK pathways

Since there is crosstalk between the PI3K/AKT/mTOR pathway and the Ras/Raf/MAPK pathway with compensatory potential, dual targeting of these two pathways is also an option. Williams et al. investigated the inhibition of both pathways in K-ras mutated pancreatic cancer cells and xenografts. While sole MEK inhibition by PD0325901 already resulted in radiosensitization and apoptosis, both effects were further enhanced by a dose of the Akt-inhibitor API-2 that was not effective on its own. Dual inhibition plus radiation also showed the most pronounced growth inhibition in a corresponding xenograft model (78). Toulany et al. demonstrated radiosensitization in K-ras mutated NSCLC cells upon PI-103 treatment but prolonged inhibition resulted in K-ras/Raf/MAPK-dependent Akt activation and loss of radiosensitization. Combining PI3K/mTOR inhibition with the MEK inhibitor PD98059 prevented the reactivation of K-ras/Raf/MAPK-dependent Akt signaling upon long-term PI-103 incubation and resulted in inhibition of DSB repair and radiosensitization (79). Using the MEK inhibitor AZD6244, Kuger et al. investigated whether additional inhibition of the MAPK pathway further enhances the radiosensitization induced by dactolisib treatment. They consistently found a radiosensitizing effect through PI3K/mTOR inhibition in lung and glioblastoma cancer cells that, however, was not increased through additional MEK inhibiton (80). Lastly, Blas et al. combined the PI3K family inhibitor buparlisib with the MEK1/2 inhibitor binimetinib in HNSCC cells. *In vitro*, both inhibitors showed a dose dependent inhibition of proliferation/viability without additional effects upon combination. None of the inhibitors, nor the combination induced radiosensitization, partly even induced radioprotection in UT-SCC-15 cells. *In vivo*, combining both inhibitors did not show any benefit in combination with irradiation and in UT-SCC-15 cells even diminished the growth delay compared to radiotherapy with either agent alone (81).

**Figure 3:**
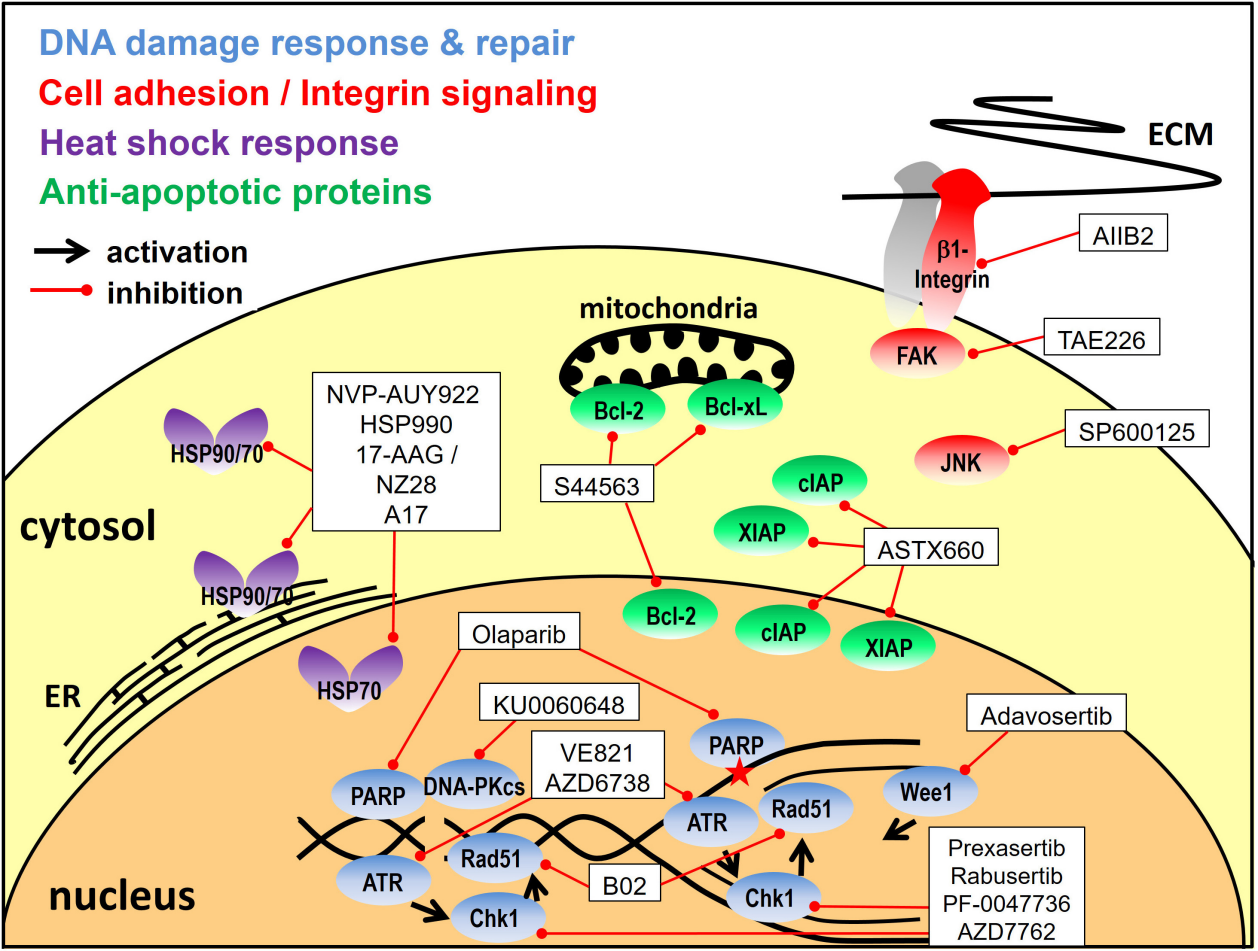
Targeting approaches other than signal transduction pathways. Depicted are the inhibitors utilized for combined molecular targeting of the DNA damage response, integrin signaling, the heat shock response or apoptosis for tumor radiosensitization and the respective target proteins. Reported inhibitor combinations are described in the text and are listed in Tables 2, 3, 4. ECM, extracellular matrix; ER, endoplasmatic reticulum.

Apart from the inhibition of signal transduction pathways a number of other strategies have been developed for tumor radiosensitization through combined molecular targeting. These include the targeting of the DNA damage response, cell adhesion molecules, the heat shock response or apoptosis, as detailed below and outlined in Figure 3.

**Table 2:** Dual targeting of DNA damage response factors.

**Targets**	**Inhibitor(s)**	**Entity**	**References**
Chk1/Wee1	LY2603618 (Rabusertib) ^*^^(disc)^ Adavosertib^**^	HNSCC (HPV+)	(95)
PARP1/ Chk1	Olaparib^**^,^***^ AZD7762^*^^(*disc*)^	Pancreatic cancer (p53 mut)	(96)
	Olaparib^**^,^***^ PF-0047736^*^^(*disc*)^	HNSCC (HPV+)	(97)
		HNSCC	(101)
PARP1/Wee1	Olaparib^**^,^***^ Adavosertib^**^	Pancreatic cancer	(98)
		Hepatocellular carcinoma	(99)
		NSCLC (K-ras mut)	(100)
		HNSCC	(101)
PARP1/ATR	Olaparib^**^,^***^ VE821^exp^	GBM	(104, 105)
PARP1/RAD51	Olaparib^*^, ^***^ B02^exp^	NSCLC, Pancreatic cancer	(106)
Chk1/2/EGFR	Prexasertib^**^ Cetuximab^**^,^***^	HNSCC	(107)
ATR/DNA-PK	AZD6738 (Ceralasertib)^*^ KU0060648^exp^	HNSCC, Colon cancer	(110)

* *Tested in clinical trials*.

** *Tested in clinical trials in combination with radiotherapy*.

*** *Approved (any clinical setting)*.

*exp, experimental; disc, discontinued*.

**Table 3:** Combined targeting approaches involving cell adhesion molecules.

**Targets**	**Inhibitor(s)**	**Entity**	**References**
β1-integrin/ EGFR	AIIB2^exp^ Cetuximab^**^,^***^	HNSCC HNSCC	(113) (115)
		Colorectal Cancer	(116)
β1-integrin/ EGFR/KEAP1/ mTOR	AIIB2^exp^ Cetuximab^**^,^***^ Everolimus^**^,^***^ ML334^exp^	HNSCC	(117)
FAK/EGFR	TAE226^exp^ Cetuximab^**^,^***^	HNSCC	(114)
β1-integrin/ c-Abl	AIIB2^exp^ Imatinib^***^	various	(118)
β1-integrin/ JNK	AIIB2^exp^ SP600125^exp^	Glioblastoma	(119)

** *Tested in clinical trials in combination with radiotherapy*.

*** *Approved (any clinical setting)*.

*exp, experimental*.

**Table 4:** Combined targeting approaches involving the heat shock response.

**Targets**	**Inhibitor(s)**	**Entity**	**References**
HSP90/HSP70	NVP-AUY922 (Luminespib)^exp^ NZ28^exp^	Lung and breast cancer	(128)
	NVP-AUY922^exp^A17 (peptide aptamer)^exp^	Lung and breast cancer	(127)
HSP90/PI3K/mTOR	NVP-AUY922^exp^PI-103^exp^	Glioblastoma, colon cancer	(130)
HSP90/PI3K	HSP990^*^^(disc)^ Buparlisib^**^	Glioma	(131)
HSP90/PARP	17-AAG (Tanespimycin)^*^^(disc)^ Olaparib^**^,^***^	Glioma	(132)

* *Tested in clinical trials*.

** *Tested in clinical trials in combination with radiotherapy*.

*** *Approved (any clinical setting)*.

*exp, experimental; disc, discontinued*.

**Table 5:** Various combined targeting approaches.

**Targets**	**Inhibitor(s)**	**Entity**	**References**
MEK/CDK4/6	Trametinib^**^,^***^ Palbociclib^**^,^***^	NSCLC (K-ras mut.)	(133)
HDAC/EGFR/ HER2	CUDC-101^*^	Pancreatic Cancer	(135)
Bcl-2/Bcl-XL	S44563^exp^	SCLC	(136)
cIAP (BIRC2)/XIAP	ASTX660^**^	HNSCC	(137)

* *Tested in clinical trials*.

** *Tested in clinical trials*.

*** *Approved (any clinical setting)*.

### Targeting the DNA Damage Response

Ionizing radiation causes DNA lesions, such as base damages, single-strand breaks, and double-strand breaks with the latter being largely responsible for cell inactivation (82). Therefore, the most obvious approach for radiosensitization is the direct targeting of the DNA damage response (DDR) and DSB repair. An integral part of the DDR are the damage induced cell cycle checkpoints in the G1, S or G2 phase, which allow additional time for DNA repair before the critical passage through mitosis where mis- or unrepaired DSBs can result in cell death due to failure in chromosome segregation (83). One of the most frequent transforming events in human cancerogenesis is the inactivation of p53. p53 mutations, the overactivation of the MDM2-controlled regulatory pathway or p53 degradation through viral oncoproteins represent the underlying mechanisms (84, 85). As p53 is essential for G1 checkpoint activation its deficiency renders affected tumor cells more dependent on S/G2 cell cycle checkpoint activation (83). Upon DNA damage these checkpoints are activated through checkpoint kinase 1 (Chk1), which is further involved in DNA repair through HR and has an impact on the stabilization of stalled replication forks and other responses to genotoxic stress during the S-phase (86). Upon activation through phosphorylation it inactivates members of the Cdc25 phosphatase family which leads to the inactivation of the cyclin-dependent kinases (CDKs) 1/2 and arrests cells in the G2 phase in response to DNA damage (87). Another kinase necessary for S and G2 checkpoint activation as well as for normal cell cycle progression is Wee1. As the direct counterpart of CDC25 phosphatases it constitutively inactivates CDK1/2 through phosphorylation and is also involved in homologous recombination (88, 89). Targeting the S- and G2-checkpoints through the inhibition of Chk1 and partly of Wee1 has been a frequently used approach for preclinical radio- or chemosensitization and was recently combined with PARP-inhibitors. The rationale is that the inhibition of PARP causes additional DNA damage especially in the S- and G2-phase through the inhibition of single-strand break (SSB) repair and PARP trapping on damaged DNA and through the subsequent collision of single strand lesions with replication forks (90–92). PARP-inhibition further impairs the alternative end-joining pathway which is also preferentially active in S- and G2 (93). These mechanisms additionally enhance the dependence on the S- and G2-checkpoints and the well described synthetic lethality of PARP-inhibition and HR deficiency (94) may further increase radiosensitization.

#### Chk1/Wee1

Focusing on cell-cycle checkpoint inhibition, Busch et al. tested the combined targeting of Chk1 (LY2603618) and Wee1 (adavosertib; AZD1775) in HPV-positive HNSCC cells because they had observed an activation of Chk1 upon Wee1 inhibition that may in part counteract the effects of sole Wee1 targeting. Analyzing proliferation, inhibition of G2 arrest and radiosensitization, they found dual targeting to be effective at profoundly reduced concentrations as compared to single agent usage. Additionally, they observed only minimal radiosensitization in p53 proficient normal human fibroblasts, thus demonstrating tumor specificity (95).

#### PARP1/Chk1

Vance et al. combined the inhibition of PARP1 through olaparib and Chk1 through AZD7762 in p53 mutant pancreatic cancer cells and observed an additive radiosensitization. The authors observed G2 checkpoint abrogation, inhibition of HR and a persistent γH2AX signal after combined inhibition of the two targets. There was no significant radiosensitization in G1-checkpoint-proficient intestinal epithelial cells, backing up the hypothesis that tumor cells harboring aberrations in p53 or other DNA damage response pathways are more selectively sensitized (96). In line with these data, Güster et al. demonstrated radiosensitization of p53 deficient HPV-positive HNSCC cells through olaparib and the Chk1-inhibitor PF-0047736, with the extent of sensitization being highest upon combined inhibition (97).

#### PARP/Wee1

Karnak et al. investigated the radiosensitizing effect of the combined inhibition of PARP1 and Wee1 through olaparib and adavosertib in pancreatic cancer cells. This dual-targeted approach is highly similar to combined PARP/Chk1-inhibition and was also associated with G2 checkpoint abrogation, inhibition of HR and persistent DNA damage. *In vitro* the combination of both inhibitors caused enhanced radiosensitization as compared to single inhibition. In *vivo*, there was no radiosensitization with olaparib alone and a moderate effect of adavosertib. Combined targeting, however, demonstrated highly significant radiosensitization (98). The same group further assessed this dual-targeting approach in hepatocellular carcinoma cells and K-ras mutant NSCLC cells, also showing an increased radiosensitization *in vitro* and *in vivo* compared to either agent alone. The authors suggested that trapping of PARP to chromatin by olaparib as well as replication stress induced through this inhibitor combination contribute to radiosensitization (99, 100). Molkentine et al. compared PARP-inhibition through niraparib plus either Wee1-inhibition through adavosertib or Chk1-inhibition through MK-8776 in an HPV-positive and an HPV-negative cell line. While both ways of S/G2-checkpoint-inhibition enhanced the radiosensitization through sole PARP-inhibition, the addition of Chk1-inhibition was more effective in the HPV-positive and of Wee1-inhibition in the HPV-negative strain. Whether these differences are generally valid for the two subentities remains to be shown in future studies (101).

#### PARP/ATR

Carruthers et al. had reported that glioblastoma stem-like cells are characterized by intrinsic replication stress, which activates the DDR and leads to radiation resistance. Ataxia telangiectasia and Rad3-related protein (ATR) is a key DDR kinase acting directly upstream of Chk1. Through Chk1 activation but also partly independent from Chk1, ATR is critically involved in replication processes, such as the stabilization of stalled replication forks, and in DSB repair pathways (102, 103). Targeting the replication stress response by a combination of olaparib and the ATR inhibitor VE821 resulted in cytotoxicity and synergistic radiosensitization, completely abolishing radioresistance (104). These data confirm results from a previous report by the same group, where the same combination resulted in greater radiosensitization than ATM inhibition in primary glioblastoma cell cultures. Radiosensitization was higher when the cells were cultured under conditions enriching the fraction of stem-like cells as compared to conditions favoring their depletion and a more differentiated state (105).

#### PARP/Rad51

Olaparib was further combined with the Rad51 inhibitor B02 and X- as well as proton-irradiation with the intention to induce HR deficiency that would synergize with PARP inhibition. Lung and pancreatic cancer cell lines were radiosensitized by the inhibitors, with the strongest effect for dual inhibition, similarly for both types of irradiation. Radiosensitization was found to be dependent on the proliferation rate, as serum deprivation reduced the effectiveness of dual targeting and in slowly proliferating PANC1 cells the combination was even less effective than sole PARP-inhibition (106).

#### Chk1/2/EGFR

The addition of the Chk1/2 inhibitor prexasertib to cetuximab and irradiation was investigated by Zeng et al. in HPV-positive and HPV-negative HNSCC cell lines. Prexasertib caused an accumulation of cells in the S-phase, the triple combination partly resulted in decreased proliferation and increased apoptosis as compared to single or double treatment (107).

#### ATR/DNA-PK

DNA-dependent protein kinase, catalytic subunit (DNA-PKcs) is well known as an essential component of the classical NHEJ pathway but is further associated with genomic stability, hypoxia, inflammatory responses, metabolism and regulation of transcription (108, 109). Hafsi et al. used combined ATR and DNA-PKcs inhibition (AZD6738, KU0060648) to radiosensitize HNSCC cells and observed an at least additive effect. A key element in this approach is that ATR inhibition interferes with cell cycle arrest and HR, whereas DNA-PKcs inhibition inhibits NHEJ. This combination therefore leaves few options for the cells to repair the radiation-induced damage in any cell cycle phase and curbs the development of resistance mechanisms. It may, however, come at the cost of tumor specificity (110).

### Targeting Cell Adhesion Molecules

Cell matrix interaction by integrins was shown to be a modulator of tumor progression, invasion, metastasis and response to therapy. β1-integrin, a member of the integrin family of cell adhesion molecules is significantly involved in tumor survival and proliferation and is associated with radio- or chemotherapy resistance (111). β1-integrin overexpression was shown in many tumor entities and its molecular targeting was found to be an effective means of radiosensitization. Integrins recruit signaling molecules to their cytoplasmic domain, mainly focal adhesion kinase (FAK) but also components of the EGFR signaling pathway, such as Erk and Akt (112). FAK is involved in proliferation, cell motility and radiation response and was found to be overexpressed or hyperphosphorylated in e.g., liver, head, and neck or breast cancer cells.

#### β1 Integrin or FAK/EGFR

Eke et al. investigated the effect of concurrent β1 integrin and EGFR targeting using the monoclonal inhibitory antibodies AIIB2 and cetuximab, respectively in head and neck cancer cells. They observed enhanced cytotoxicity and radiosensitization upon combined inhibition in 8 out of 10 cell lines and, in line with that, enhanced survival in a xenograft model of a responder cell line (113). FAK was shown to mediate the effects of β1 integrin targeting in line with previous reports of the same group that had shown dual inhibition of EGFR (cetuximab, siRNA) and FAK (TAE226, siRNA) to achieve a stronger radiosensitizing effect in HNSCCs than either inhibitor alone (114). Zscheppang et al. further investigated single and dual β1-integrin/EGFR targeting using AIIB2 & cetuximab in sphere-forming HNSCC cells based on the concept that tumor initiating cells are enriched in spheres. Sphere-forming cells were found to be resistant to this targeting approach and future work is warranted to understand the mechanisms and relevance of this finding (115). In another report, the same dual β1-integrin/EGFR inhibition approach, as well as KRAS or BRAF depletion and 5-FU-treatment failed to modulate the radiosensitivity of colorectal carcinoma cells (116).

Recently, a screen for predictive biomarkers for the dual β1-integrin/EGFR targeting approach showed different mutational profiles of responding and non-responding cells and suggested some proteins as potential resistance factors. Using an RNAi screen and pharmacological inhibition (ML334, everolimus) Kelch like ECH associated protein 1 (KEAP1) and mTOR were identified as druggable targets for radiosensitization in combination with β1-integrin/EGFR targeting (117).

#### β1-Integrin/c-Abl

C-Abl is a tyrosine kinase found to be hyperphosphorylated upon β1-integrin inhibition. Therefore, dual β1-integrin (AIIB2) and c-Abl (imatinib) targeting was tested in a panel of tumor cell lines from various entities, where a cell line dependent cytotoxicity and enhancement or induction of radiosensitivity was observed as compared to single treatment in a subgroup of the panel. Radiosensitization was accompanied by altered expression of DSB repair proteins KU70 and NBS1 and was associated with reduced DSB repair (118).

#### β1-Integrin/JNK

Vehlow et al. identified the c-Jun N-terminal kinase (JNK), a known stress mediator, to mediate bypass signaling after β1-integrin-inhibition in established glioblastoma cell lines, as well as stem-like and patient-derived glioblastoma cells. Dual β1-integrin/JNK inhibition through AIIB2 and the JNK inhibitor SP600125 *in vitro* and *in vivo* resulted in a superior effect when combined with radiation as compared to single inhibition, i.e., increasing the median survival of orthotopic, radiochemotherapy-treated GBM mice. *In vitro* the authors observed defects in DNA repair associated with chromatin changes, enhanced ATM phosphorylation, and prolonged G2/M cell cycle arrest as the underlying mechanism of radiosensitization (119).

### Targeting the Heat-Shock Response

Heat shock proteins (HSPs) are a group of proteins with enhanced expression in response to various kinds of stresses, such as hyperthermia, infections, heavy metals, or oxidative stress (120). As molecular chaperones they assist their substrate proteins, termed clients, in acquiring or recovering their functional three dimensional fold, a process especially important under stressed conditions. Furthermore they assist the binding of ligands to their targets and the assembly of multiprotein complexes and they are potent inhibitors of apoptosis (121, 122). HSP70 and HSP90 proteins represent two important, druggable HSP families with actually hundreds of client proteins making their molecular targeting a biologically complex approach with numerous possible subsequent effects. The inhibition of both HSP70 and HSP90 are being tested for cancer therapy because of their especially high expression levels in tumors. Enhanced expression is believed to be necessary because in tumor cells proteostasis is permanently challenged by tumor cell metabolism, oxidative stress, dysregulated protein expression and the expression of mutant (onco) proteins, which may be less stable and require more assistance from the chaperone machinery (123). A prominent example for the latter is the stabilization of mutant, gain of function p53 variants through HSP90 (124).

#### HSP90/HSP70

It was shown that the inhibition of Hsp90 compromises DNA repair after irradiation and enhances tumor cell radiosensitivity (125, 126). However, HSP90 inhibition also leads to the activation of the transcription factor Heat Shock Factor 1 (HSF 1). HSF-1 is inactivated when bound by HSPs and becomes active upon release, e.g., upon HSP-inhibition or under stressed conditions, in order to adjust the HSP-expression level to the chaperone demand of the cell (120). Therefore, targeting HSP90 can enhance the expression of Hsp70, which may partly antagonize the effects of HSP90-inhibition. This led to the dual targeting approach of Schilling et al. in which HSP70 inhibition through the peptide aptamer A17 failed to significantly radiosensitize lung and breast cancer cells on its own but augmented the radiosensitizing effect of the Hsp90 inhibitor NVP-AUY922. The authors suggested that increased levels of DNA double-strand breaks and enhanced G2/M arrest are involved in cell death after combined treatment and radiation (127). In a previous work the same group had already shown similar results in which addition of NVP-AUY922 allowed for a reduction in the concentration of the HSP70 inhibitor NZ28 to 1/10 to 1/20 to still achieve the same radiosensitization (128).

#### HSP90/PI3K/mTOR

Following the same concept as dual HSP70/90-inhibition the PI3K/mTOR inhibitor PI-103, which had previously been shown to suppress the up-regulation of Hsp70 (129), was combined with Hsp90-inhibition through NVP-AUY922. Adding both inhibitors 3 h prior to irradiation followed by 24 h of culture moderately enhanced the radiosensitizing effect. The authors supposed a down regulation of PI3K and ERK pathways, increased DNA damage, and a pronounced G2/M arrest as possible causative factors. Interestingly, using another treatment schedule, adding the inhibitor 24 h before irradiation slightly reduces the radiosensitizing effect of the HSP90 inhibitor. They considered a reactivation of the PI3K/MAPK pro-survival pathway and an increased G1 arrest at the moment of irradiation and better DNA repair to cause these controversial observations. These findings underline the importance of IR-drug scheduling (130).

In human glioma cells Wachsberger et al. combined the PI3K inhibitor Buparlisib (BKM120) with the HSP90 inhibitor HSP990, which resulted in downregulation of the AKT pathway and induction of apoptosis. *In vitro* from a panel of four cell lines only U373MG showed a profound radiosensitization after dual targeting as compared to single inhibition. Still, *in vivo*, U87MG showed a more pronounced tumor growth delay compared to single inhibition with and without the combination with irradiation (131).

#### HSP90/PARP1

Also targeting human glioma cells Dungey et al. combined the inhibition of PARP through olaparib with the Hsp90 inhibitor 17-AAG. The rationale behind is that Hsp90-inhibition decreases HR, which is needed to repair replication-associated DSB generated through PARP inhibition. They observed a downregulation of Rad51 and BRCA2 protein levels and inhibition of HR upon HSP90 inhibition. Combined treatment resulted in additive radiosensitization in proliferating cells. Since the authors did not observe radiosensitization through HSP90 inhibition in non-tumor control cells and had previously described olaparib-mediated radiosensitization to be replication-dependent, they expect an enhancement of the therapeutic ratio by taking advantage of the non-dividing state of normal brain tissue (132).

### Other Approaches

#### MEK/Cyclin Dependent Kinases (CDKs)

Tao et al. observed that in Kras-mutant NSCLC cells, the inhibition of MEK through trametinib resulted in p16 expression and reduced phosphorylation and therefore activation of the tumorsuppressor RB in the cell line most sensitive toward both sole MEK-inhibition and MEK-inhibition induced radiosensitization. Likewise, activation of RB through CDK4/6-inhibition through palbociclib sensitized the more resistant cells to MEK-inhibition and resulted in enhanced radiation sensitivity as compared to single treatment. Dual targeting plus irradiation was also most effective in a xenograft model (133).

#### Targeting Histone Deacetylases/HER Family Receptors

Histone deacetylase inhibitors are a heterogeneous group of epigenetic therapeutics, which a.o. interfere with DNA damage signaling and repair (134). Moertl et al. have compared the radiosensitization of pancreatic cancer cells through the HDAC inhibitor SAHA and the multi target inhibitor CUDC-101, which, besides HDAC, also targets EGFR and HER2 (135). They observed reduced proliferation and clonogenic survival and increased apoptosis with reduced expression of the antiapoptotic proteins XIAP and survivin with both inhibitors. While the multi target inhibitor was identified as the more potent radiosensitizer, no clues can presently be drawn regarding a synergistic mechanism of HDAC and EGFR/HER2 targeting since no combined treatment of SAHA and HER family receptor inhibition was performed.

#### Targeting Anti-apoptotic Proteins

We further identified two studies, which followed a strategy of inhibiting two anti-apoptotic proteins. In the first study, Bcl-2 and Bcl-XL, two members of the anti-apoptotic fraction of the Bcl-2 family of mitochondrial membrane proteins, were inhibited using the dual inhibitor S44563. Upon targeting plus irradiation the authors observed an enhanced sub-G1 fraction and caspase 3 cleavage as compared to single treatment. In clonogenic assays they further observed radiosensitization and a slight growth delay in xenograft models upon inhibition plus radiation. Interestingly, treatment was most effective when the inhibitor was added after completion of fractionated radiation, highlighting the importance of the optimal sequence of modalities (136). Another approach utilized a novel antagonist of the E3 ubiquitin ligases cIAP and XIAP (cellular inhibitor of apoptosis protein, X-linked inhibitor of apoptosis protein), ASTX660. *In vitro*, the inhibitor sensitized subsets of HPV-negative and HPV-positive HNSCC cell lines to TNF family death ligands TNFα and TRAIL, which involved a reactivation of p53 in the HPV-positive strains. In HPV-positive and -negative human HNSCC xenografts the authors observed significantly delayed growth when the dual inhibitor was combined with radiation, which was attenuated by anti-TNFα pretreatment blockade (137).

## Clinical Trials

Despite a plethora of positive preclinical data, molecular targeting for tumor radiosensitization is not yet a valid treatment option in the clinic, but a considerable number of clinical trials is testing targeting approaches in combination with (chemo)radiation. However, when searching for trials with combined molecular targeting for radiosensitization in the narrow sense, we only found two running studies combining two inhibitors with sole radiotherapy, both in HNSCC. One trial is testing the combination of cetuximab and the CDK4/6 inhibitor palbociclib, a combination, for which we did not find a preceding preclinical evaluation but only a similar approach combining MEK-inhibition plus palbociclib (133) (NCT03024489). The other trial compares the dual targeting of EGFR and Chk1 through cetuximab and prexasertib vs. the combination of cisplatin and prexasertib (NCT02555644). Since this design does not include single inhibition or standard treatment but uses the non-approved inhibitor prexasertib in both arms, it may become difficult to finally estimate to what extent this dual targeting approach may increase radiation sensitivity.

In the following we present a selection of relevant publications reporting results from clinical trials using combined molecular targeting and (mostly chemo-)radiation. A common approach is the combination of chemoradiation, inhibition of signal transduction pathways and VEGFR-inhibition. However, while these combined inhibitor approaches clearly cover anticipated effectiveness through dual targeting, the purpose of radiosensitization is less in focus than the idea of achieving additive effects through repression of angiogenesis. Especially the combination of chemoradiation, EGFR-inhibition and the anti-VEGF antibody bevacizumab has been tested in different entities but efficacy so far appears limited: In HNSCC, the addition of bevacizumab to radiation, cetuximab and pemetrexed was reported to increase toxicity without an apparent improvement in efficacy (138). Similarly, the combination of (chemo)radiation, bevacizumab and erlotinib did not result in a survival benefit but demonstrated targeted-agent specific toxicity in esophageal cancer (139). Along the same line, adding erlotinib to chemoradiation and bevacizumab did not show efficacy but induced esophageal toxicity in NSCLC (140) and the addition of the EGFR/VEGFR inhibitor vandetanib did not prolong survival in a phase II study of glioblastoma (141). In contrast to these clearly negative results, encouraging responses have been reported for the addition of erlotinib to neoadjuvant chemoradiation plus bevacizumab in phase I trials of rectal cancer, which warrant further investigation in larger studies (142, 143). Clinical results have also been reported for the inhibition of EGFR and HER2 in HNSCC through lapatinib. Addition to primary chemoradiation plus lapatinib maintenance resulted in increased 6-month complete response rates and progression-free survival as compared to placebo in a phase II study (144). However, in a similar design in the setting of adjuvant chemoradiation after surgery, lapatinib did not result in any efficacy benefits but additional toxicity in a large phase III trial of 688 patients (145).

At present, molecular targeting is often added to current chemoradiation regimes to increase efficacy and trials are mostly in early clinical development. It is therefore not surprising that combined targeting approaches are still quite rare and often based on dual specific inhibitors, which may be easier to implement than inhibitor combinations for which toxicity data may still be lacking, even without radiotherapy.

## Discussion

Molecular targeting approaches for tumor radiosensitization have been investigated for two decades (146–149), but their implementation into the clinic has proven extremely difficult. As outlined above, the only molecular targeting agent that is approved in the curative setting in combination with radiotherapy is the anti-EGFR-antibody cetuximab in HNSCC, and considerable doubts exist regarding its efficacy (150). The various approaches described in this review aimed to achieve a meaningful radiosensitization through combined inhibition of two or more targets, mostly with the aim of a more complete pathway inhibition or the suppression of compensatory mechanisms, partly with similarities to the concept of synthetic lethality (Supplementary Figure 1). The diversity of the approaches reflects the heterogeneity of radiosensitization strategies although some additional emerging concepts, such as interference with NAD^+^-, glucose- or mitochondrial metabolism (151, 152) or the eradication of cancer stem cells (153, 154) were not identified in our search for combined targeting approaches. While molecular targeting is also increasingly being considered as a strategy to enhance the efficacy of particle irradiation (155), we only identified one such publication, which reported the effect of PARP and Rad51 inhibition when added to proton (and photon) irradiation (106).

With 49 identified publications, the combined targeting of classical kinase dependent signal transduction pathways, such as EGFR, MAPK, or PI3K/AKT/mTOR signaling is the most exhaustively studied approach. The underlying rationale is that tumor cells often rely on the hyperactivation of these kinases to drive key mechanisms such as proliferation, survival and, to some extent, DNA repair (“oncogene addiction”). This should make them more sensitive to kinase inhibitors than normal tissue, providing some tumor specificity. What remains a major challenge is the choice of pathway inhibition for individual tumors, which requires reliable biomarkers. Unfortunately, the commonly analyzed kinase expression level is a poor surrogate for actual kinase activity, as we have recently demonstrated for EGFR activation in HNSCC (156). Keeping this in mind, it will be crucial to establish robust markers of aberrantly high activity, e.g., the detection of activating mutations, protein phosphorylation levels, or functional measurements. Given the identification of an overactive pathway, dual inhibition may be an appropriate way to achieve highly effective inhibition or to avoid bypass signaling through compensatory pathways or mutations of downstream pathway members. To what extent and in which setting a more effective inhibition of signal transduction pathways will subsequently also translate into a clinically meaningful radiosensitization and finally enhanced patient survival remains to be shown.

A major advantage for the targeting of DDR components is their direct involvement in radiation-induced DNA repair. On one hand, this makes it likely that a majority of tumors will be affected. On the other hand, specificity can be a major issue, as normal cells utilize the same pathways for DNA damage recognition, processing and repair. Nine of the 13 studies identified in this field combined PARP- and S/G2 phase checkpoint-inhibition. This approach is partly based on the model of synthetic lethality (see Supplementary Figure 1), which has been described for PARP inhibition and HR deficiency (157), as the inhibition of Chk1, Wee1, and ATR was reported to compromise HR (89, 158–160). The same concept applies to the direct targeting of the central HR factor Rad51 combined with PARP inhibition (106) and in part HSP90 inhibition plus PARP inhibition (132). As HR is only active in the S/G2 phase, some degree of tumor specificity can be expected because normal tissue cells mostly do not proliferate and, in contrast to the majority of tumor cells, are p53 proficient and therefore able to arrest in the G1 phase after irradiation. Additional S/G2 phase-derived DNA damage through PARP inhibition is likely to further increase the dependence on S/G2 arrest, which may further be fostered by oncogenic replication stress.

Similar characteristics, i.e., high pathway activity and tumor cells‘ reliance also motivate the targeting of adhesion molecules [e.g., high expression of focal adhesion signaling receptors (112)] or the heat shock response [e.g., proteostatic control of instable mutants (123)] in order to achieve tumor specificity. Again, the additional inhibition of compensatory factors provides a main rationale for dual targeting approaches.

The tailored use of molecular targeting agents based on individual tumor characteristics is referred to as precision oncology. Apart from enhancing efficacy and thereby cure rates, molecular targeting is also expected to reduce toxicity, in case it can replace chemotherapy. It has to be noted, however, that the use of targeting agents can result in considerable side effects, which can of course be more severe and difficult to predict when agents are combined and added to (chemo-) radiotherapy. For example, EGFR inhibition frequently causes skin rash and diarrhea (161), which can be especially severe in the radiation field, when the effects add up with (chemo-) radiation induced erythema/mucositis (162). In a phase 3 study for HNSCC the addition of cetuximab to cisplatin-based chemoradiation resulted in considerably more grade 3/4 mucositis and rash and hence higher rates of interruptions in radiation therapy without achieving any clinical benefit (8). As further examples, combining bevacizumab with concomitant radiotherapy can lead to decreased wound healing (163, 164) and, in patients with lung cancer, to fistula formation (165), and the Chk1 inhibitors LY2603618 and ADZ7762 increased the risk of severe thromboembolic events (166) and cardiac side effects (167), respectively, in part when combined with chemotherapy. At present, preclinical data on tumor radiosensitization hardly ever contain thorough *in vivo* analyses of side effects other than weight loss and inspection of the skin/mucosa in the radiation field. Future approaches should therefore not only focus on the identification of the most efficacious radiosensitization but also more deeply on the safety of a possible clinical translation. Detailed *in vivo* studies on systemic as well as in-field toxicity may help design the most promising clinical trials and achieve better clinical outcomes.

In conclusion, dual targeting for tumor radiosensitization has shown promising results in pre-clinical studies. The way to proceed toward a substantial future clinical benefit requires convincing *in vitro* mechanistic studies that should ideally include predictive biomarkers, with the results substantiated in a relevant number of adequate model systems, such as cell lines and (patient derived) xenografts, but possibly also tumor stem cell cultures as well as *ex vivo* cultured tumor tissues. The most promising combined targeting approaches should be thoroughly inspected for treatment efficacy and safety based on normal tissue toxicity *in vivo*. Such a concept should lead to the identification of effective targeting strategies for subsets of tumors, based on reliable predictive biomarkers to provide the best possible preclinical rationale to allow clinicians to implement the most appropriate combined targeting strategies in well-designed clinical trials.

## Author Contributions

KH and TR: idea, literature search, and preparation of tables and figures. All authors were involved in the preparation of the manuscript.

## Conflict of Interest

The authors declare that the research was conducted in the absence of any commercial or financial relationships that could be construed as a potential conflict of interest.

## References

[1] Blanchard P Baujat B Holostenco V Bourredjem A Baey C Bourhis J. Meta-analysis of chemotherapy in head and neck cancer (MACH-NC): a comprehensive analysis by tumour site. Radiother Oncol. (2011) 100:33–40. 10.1016/j.radonc.2011.05.036 21684027

[2] Bourhis J Sire C Graff P Gregoire V Maingon P Calais G. Concomitant chemoradiotherapy versus acceleration of radiotherapy with or without concomitant chemotherapy in locally advanced head and neck carcinoma (GORTEC 99-02): an open-label phase 3 randomised trial. Lancet Oncol. (2012) 13:145–53. 10.1016/S1470-2045(11)70346-1 22261362

[3] Dasari S Tchounwou PB. Cisplatin in cancer therapy: molecular mechanisms of action. Eur J Pharmacol. (2014) 740:364–78. 10.1016/j.ejphar.2014.07.025 25058905 PMC4146684

[4] Bonner JA Harari PM Giralt J Azarnia N Shin DM Cohen RB. Radiotherapy plus cetuximab for squamous-cell carcinoma of the head and neck. N Engl J Med. (2006) 354:567–78. 10.1056/NEJMoa053422 16467544

[5] Petrelli F Coinu A Riboldi V Borgonovo K Ghilardi M Cabiddu M Concomitant platinum-based chemotherapy or cetuximab with radiotherapy for locally advanced head and neck cancer: a systematic review and meta-analysis of published studies. Oral Oncol. (2014) 50:1041–8. 10.1016/j.oraloncology.2014.08.005 25176576

[6] Magrini SM Buglione M Corvo R Pirtoli L Paiar F Ponticelli P. Cetuximab and radiotherapy versus cisplatin and radiotherapy for locally advanced head and neck cancer: a randomized phase II trial. J Clin Oncol. (2016) 34:427–35. 10.1200/JCO.2015.63.1671 26644536

[7] Beckham TH Barney C Healy E Wolfe AR Branstetter A Yaney A. Platinum-based regimens versus cetuximab in definitive chemoradiation for human papillomavirus-unrelated head and neck cancer. Int J Cancer. (2019) 147:107–15. 10.1002/ijc.32736 31609479

[8] Ang KK Zhang Q Rosenthal DI Nguyen-Tan PF Sherman EJ Weber RS Randomized phase III trial of concurrent accelerated radiation plus cisplatin with or without cetuximab for stage III to IV head and neck carcinoma: RTOG 0522. J Clin Oncol. (2014) 32:2940–50. 10.1200/JCO.2013.53.5633 25154822 PMC4162493

[9] Gillison ML Trotti AM Harris J Eisbruch A Harari PM Adelstein DJ. Radiotherapy plus cetuximab or cisplatin in human papillomavirus-positive oropharyngeal cancer. (NRG Oncology RTOG 1016): a randomised, multicentre, non-inferiority trial. Lancet. (2019) 393:40–50. 10.1016/S0140-6736(18)32779-X 30449625 PMC6541928

[10] Mehanna H Robinson M Hartley A Kong A Foran B Fulton-Lieuw T. Radiotherapy plus cisplatin or cetuximab in low-risk human papillomavirus-positive oropharyngeal cancer. (De-ESCALaTE HPV): an open-label randomised controlled phase 3 trial. Lancet. (2019) 393:51–60. 10.1016/S0140-6736(18)32752-1 30449623 PMC6319250

[11] Rosenthal DI Harari PM Giralt J Bell D Raben D Liu J Association of human papillomavirus and p16 status with outcomes in the IMCL-9815 phase III registration trial for patients with locoregionally advanced oropharyngeal squamous cell carcinoma of the head and neck treated with radiotherapy with or without cetuximab. J Clin Oncol. (2016) 34:1300–8. 10.1200/JCO.2015.62.5970 26712222 PMC5070577

[12] Morgan MA Parsels LA Maybaum J Lawrence TS. Improving the efficacy of chemoradiation with targeted agents. Cancer Discov. (2014) 4:280–91. 10.1158/2159-8290.CD-13-0337 24550033 PMC3947675

[13] Meyn RE Munshi A Haymach JV Milas L Ang KK. Receptor signaling as a regulatory mechanism of DNA repair. Radiother Oncol. (2009) 92:316–22. 10.1016/j.radonc.2009.06.031 19615770 PMC2754282

[14] Toulany M. Targeting DNA double-strand break repair pathways to improve radiotherapy response. Genes. (2019) 10:25. 10.3390/genes10010025 30621219 PMC6356315

[15] Liu Q Yu S Zhao W Qin S Chu Q Wu K. EGFR-TKIs resistance via EGFR-independent signaling pathways. Mol Cancer. (2018) 17:53. 10.1186/s12943-018-0793-1 29455669 PMC5817859

[16] Bogdan S Klambt C. Epidermal growth factor receptor signaling. Curr Biol. (2001) 11:R292–5. 10.1016/S0960-9822(01)00167-1 11369216

[17] Normanno N De Luca A Bianco C Strizzi L Mancino M Maiello MR. Epidermal growth factor receptor. (EGFR) signaling in cancer. Gene. (2006) 366:2–16. 10.1016/j.gene.2005.10.018 16377102

[18] Bianco R Gelardi T Damiano V Ciardiello F Tortora G. Rational bases for the development of EGFR inhibitors for cancer treatment. Int J Biochem Cell Biol. (2007) 39:1416–31. 10.1016/j.biocel.2007.05.008 17596994

[19] Mendelsohn J Baselga J Epidermal growth factor receptor targeting in cancer. Semin Oncol. (2006) 33:369–85. 10.1053/j.seminoncol.2006.04.003 16890793

[20] Fukutome M Maebayashi K Nasu S Seki K Mitsuhashi N. Enhancement of radiosensitivity by dual inhibition of the HER family with ZD1839. (“Iressa”) and trastuzumab. (“Herceptin”). Int J Radiat Oncol Biol Phys. (2006) 66:528–36. 10.1016/j.ijrobp.2006.05.036 16965995

[21] Zhou H Kim YS Peletier A Mccall W Earp HS Sartor CI. Effects of the EGFR/HER2 kinase inhibitor GW572016 on EGFR- and HER2-overexpressing breast cancer cell line proliferation, radiosensitization, and resistance. Int J Radiat Oncol Biol Phys. (2004) 58:344–52. 10.1016/j.ijrobp.2003.09.046 14751502

[22] Sambade MJ Camp JT Kimple RJ Sartor CI Shields JM. Mechanism of lapatinib-mediated radiosensitization of breast cancer cells is primarily by inhibition of the Raf>MEK>ERK mitogen-activated protein kinase cascade and radiosensitization of lapatinib-resistant cells restored by direct inhibition of MEK. Radiother Oncol. (2009) 93:639–44. 10.1016/j.radonc.2009.09.006 19853943 PMC2799330

[23] Kimple RJ Vaseva AV Cox AD Baerman KM Calvo BF Tepper JE. Radiosensitization of epidermal growth factor receptor/HER2-positive pancreatic cancer is mediated by inhibition of Akt independent of ras mutational status. Clin Cancer Res. (2010) 16:912–23. 10.1158/1078-0432.CCR-09-1324 20103665 PMC2818631

[24] Sambade MJ Kimple RJ Camp JT Peters E Livasy CA Sartor CI. Lapatinib in combination with radiation diminishes tumor regrowth in HER2+ and basal-like/EGFR+ breast tumor xenografts. Int J Radiat Oncol Biol Phys. (2010) 77:575–81. 10.1016/j.ijrobp.2009.12.063 20457354 PMC2976524

[25] Yu T Cho BJ Choi EJ Park JM Kim DH Kim IA. Radiosensitizing effect of lapatinib in human epidermal growth factor receptor 2-positive breast cancer cells. Oncotarget. (2016) 7:79089–100. 10.18632/oncotarget.12597 27738326 PMC5346700

[26] Paldor I Abbadi S Bonne N Ye X Rodriguez FJ Rowshanshad D. The efficacy of lapatinib and nilotinib in combination with radiation therapy in a model of NF2 associated peripheral schwannoma. J Neurooncol. (2017) 135:47–56. 10.1007/s11060-017-2567-9 28735458

[27] Mu Y Sun D. Lapatinib, a dual inhibitor of epidermal growth factor receptor (EGFR) and HER-2, enhances radiosensitivity in mouse bladder tumor line-2 (MBT-2) cells *in vitro* and *in vivo*. Med Sci Monit. (2018) 24:5811–9. 10.12659/MSM.909865 30125265 PMC6113922

[28] Huang S Li C Armstrong EA Peet CR Saker J Amler LC. Dual targeting of EGFR and HER3 with MEHD7945A overcomes acquired resistance to EGFR inhibitors and radiation. Cancer Res. (2013) 73:824–33. 10.1158/0008-5472.CAN-12-1611 23172311 PMC7254871

[29] Li C Huang S Armstrong EA Francis DM Werner LR Sliwkowski MX. Antitumor effects of MEHD7945A, a dual-specific antibody against EGFR and HER3, in combination with radiation in lung and head and neck cancers. Mol Cancer Ther. (2015) 14:2049–59. 10.1158/1535-7163.MCT-15-0155 26141946

[30] Van Der Veeken J Oliveira S Schiffelers RM Storm G Van Bergen En Henegouwen PM Roovers RC. Crosstalk between epidermal growth factor receptor- and insulin-like growth factor-1 receptor signaling: implications for cancer therapy. Curr Cancer Drug Targets. (2009) 9:748–60. 10.2174/156800909789271495 19754359

[31] Matsumoto F Valdecanas DN Mason KA Milas L Ang KK Raju U. The impact of timing of EGFR and IGF-1R inhibition for sensitizing head and neck cancer to radiation. Anticancer Res. (2012)32:3029–35. 22843870

[32] Wang Y Yuan JL Zhang YT Ma JJ Xu P Shi CH. Inhibition of both EGFR and IGF1R sensitized prostate cancer cells to radiation by synergistic suppression of DNA homologous recombination repair. PLoS ONE. (2013) 8:e68784. 10.1371/journal.pone.0068784 23950876 PMC3741308

[33] Li P Veldwijk MR Zhang Q Li ZB Xu WC Fu S. Co-inhibition of epidermal growth factor receptor and insulin-like growth factor receptor 1 enhances radiosensitivity in human breast cancer cells. BMC Cancer. (2013) 13:297. 10.1186/1471-2407-13-297 23777562 PMC3697997

[34] Benavente S Huang S Armstrong EA Chi A Hsu KT Wheeler DL. Establishment and characterization of a model of acquired resistance to epidermal growth factor receptor targeting agents in human cancer cells. Clin Cancer Res. (2009) 15:1585–92. 10.1158/1078-0432.CCR-08-2068 19190133 PMC2903727

[35] Karapetis CS Khambata-Ford S Jonker DJ O'callaghan CJ Tu D Tebbutt NC. K-ras mutations and benefit from cetuximab in advanced colorectal cancer. N Engl J Med. (2008) 359:1757–65. 10.1056/NEJMoa0804385 18946061

[36] Bokemeyer C Bondarenko I Hartmann JT De Braud F Schuch G Zubel A. Efficacy according to biomarker status of cetuximab plus FOLFOX-4 as first-line treatment for metastatic colorectal cancer: the OPUS study. Ann Oncol. (2011) 22:1535–46. 10.1093/annonc/mdq632 21228335

[37] Bonner JA Trummell HQ Bonner AB Willey CD Bredel M Yang ES. Enhancement of Cetuximab-Induced Radiosensitization by JAK-1 Inhibition. BMC Cancer. (2015) 15:673. 10.1186/s12885-015-1679-x 26458879 PMC4603644

[38] Eke I Schneider L Forster C Zips D Kunz-Schughart LA Cordes N. EGFR/JIP-4/JNK2 signaling attenuates cetuximab-mediated radiosensitization of squamous cell carcinoma cells. Cancer Res. (2013) 73:297–306. 10.1158/0008-5472.CAN-12-2021 23074283

[39] Zhuang H Bai J Chang JY Yuan Z Wang P. MTOR inhibition reversed drug resistance after combination radiation with erlotinib in lung adenocarcinoma. Oncotarget. (2016) 7:84688–94. 10.18632/oncotarget.12423 27713162 PMC5356691

[40] Ellis LM Hicklin DJ. VEGF-targeted therapy: mechanisms of anti-tumour activity. Nat Rev Cancer. (2008) 8:579–91. 10.1038/nrc2403 18596824

[41] Goel HL Mercurio AM. VEGF targets the tumour cell. Nat Rev Cancer. (2013) 13:871–82. 10.1038/nrc3627 24263190 PMC4011842

[42] Huang SM Harari PM. Modulation of radiation response after epidermal growth factor receptor blockade in squamous cell carcinomas: inhibition of damage repair, cell cycle kinetics, and tumor angiogenesis. Clin Cancer Res. (2000) 6:2166–74. 10873065

[43] Tabernero J. The role of VEGF and EGFR inhibition: implications for combining anti-VEGF and anti-EGFR agents. Mol Cancer Res. (2007) 5:203–20. 10.1158/1541-7786.MCR-06-0404 17374728

[44] Bozec A Formento P Lassalle S Lippens C Hofman P Milano G. Dual inhibition of EGFR and VEGFR pathways in combination with irradiation: antitumour supra-additive effects on human head and neck cancer xenografts. Br J Cancer. (2007) 97:65–72. 10.1038/sj.bjc.6603791 17592499 PMC2359670

[45] Bozec A Sudaka A Fischel JL Brunstein MC Etienne-Grimaldi MC Milano G. Combined effects of bevacizumab with erlotinib and irradiation: a preclinical study on a head and neck cancer orthotopic model. Br J Cancer. (2008) 99:93–9. 10.1038/sj.bjc.6604429 18577994 PMC2453013

[46] Bozec A Sudaka A Toussan N Fischel JL Etienne-Grimaldi MC Milano G. Combination of sunitinib, cetuximab and irradiation in an orthotopic head and neck cancer model. Ann Oncol. (2009) 20:1703–7. 10.1093/annonc/mdp070 19542251

[47] Bozec A Etienne-Grimaldi MC Fischel JL Sudaka A Toussan N Formento P. The mTOR-targeting drug temsirolimus enhances the growth-inhibiting effects of the cetuximab-bevacizumab-irradiation combination on head and neck cancer xenografts. Oral Oncol. (2011) 47:340–4. 10.1016/j.oraloncology.2011.02.020 21421338

[48] Bozec A Lassalle S Gugenheim J Fischel JL Formento P Hofman P. Enhanced tumour antiangiogenic effects when combining gefitinib with the antivascular agent ZD6126. Br J Cancer. (2006) 95:722–8. 10.1038/sj.bjc.6603308 16940984 PMC2360508

[49] Shibuya K Komaki R Shintani T Itasaka S Ryan A Jurgensmeier JM. Targeted therapy against VEGFR and EGFR with ZD6474 enhances the therapeutic efficacy of irradiation in an orthotopic model of human non-small-cell lung cancer. Int J Radiat Oncol Biol Phys. (2007) 69:1534–43. 10.1016/j.ijrobp.2007.07.2350 17889445 PMC2151850

[50] Oehler-Janne C Jochum W Riesterer O Broggini-Tenzer A Caravatti G Vuong V. Hypoxia modulation and radiosensitization by the novel dual EGFR and VEGFR inhibitor AEE788 in spontaneous and related allograft tumor models. Mol Cancer Ther. (2007) 6:2496–504. 10.1158/1535-7163.MCT-07-0253 17876047

[51] Wachsberger PR Lawrence YR Liu Y Daroczi B Xu X Dicker AP Epidermal growth factor receptor expression modulates antitumor efficacy of vandetanib or cediranib combined with radiotherapy in human glioblastoma xenografts. Int J Radiat Oncol Biol Phys. (2012) 82:483–91. 10.1016/j.ijrobp.2010.09.019 21095630

[52] Horn D Hess J Freier K Hoffmann J Freudlsperger C. Targeting EGFR-PI3K-AKT-mTOR signaling enhances radiosensitivity in head and neck squamous cell carcinoma. Expert Opin Ther Targets. (2015) 19:795–805. 10.1517/14728222.2015.1012157 25652792

[53] Wise HM Hermida MA Leslie NR. Prostate cancer, PI3K, PTEN and prognosis. Clin Sci. (2017) 131:197–210. 10.1042/CS20160026 28057891

[54] Sobhani N Roviello G Corona SP Scaltriti M Ianza A Bortul M. The prognostic value of PI3K mutational status in breast cancer: a meta-analysis. J Cell Biochem. (2018) 119:4287–92. 10.1002/jcb.26687 29345357 PMC5995110

[55] Marquard FE Jücker M. PI3K/AKT/mTOR signaling as a molecular target in head and neck cancer. Biochem Pharmacol. (2019) 172:113729. 10.1016/j.bcp.2019.113729 31785230

[56] Miyahara H Yadavilli S Natsumeda M Rubens JA Rodgers L Kambhampati M The dual mTOR kinase inhibitor TAK228 inhibits tumorigenicity and enhances radiosensitization in diffuse intrinsic pontine glioma. Cancer Lett. (2017) 400:110–6. 10.1016/j.canlet.2017.04.019 28450157 PMC5569904

[57] Liu ZG Tang J Chen Z Zhang H Wang H Yang J. The novel mTORC1/2 dual inhibitor INK128 enhances radiosensitivity of breast cancer cell line MCF-7. Int J Oncol. (2016) 49:1039–45. 10.3892/ijo.2016.3604 27574017

[58] Hayman TJ Kramp T Kahn J Jamal M Camphausen K Tofilon PJ. Competitive but not allosteric mTOR kinase inhibition enhances tumor cell radiosensitivity. Transl Oncol. (2013) 6:355–62. 10.1593/tlo.13163 23730416 PMC3660805

[59] Kahn J Hayman TJ Jamal M Rath BH Kramp T Camphausen K. The mTORC1/mTORC2 inhibitor AZD2014 enhances the radiosensitivity of glioblastoma stem-like cells. Neuro Oncol. (2014) 16:29–37. 10.1093/neuonc/not139 24311635 PMC3870843

[60] Yu CC Hung SK Lin HY Chiou WY Lee MS Liao HF. Targeting the PI3K/AKT/mTOR signaling pathway as an effectively radiosensitizing strategy for treating human oral squamous cell carcinoma *in vitro* and *in vivo*. Oncotarget. (2017) 8:68641–53. 10.18632/oncotarget.19817 28978144 PMC5620284

[61] Mukherjee B Tomimatsu N Amancherla K Camacho CV Pichamoorthy N Burma S. The dual PI3K/mTOR inhibitor NVP-BEZ235 is a potent inhibitor of ATM- and DNA-PKCs-mediated DNA damage responses. Neoplasia. (2012) 14:34–43. 10.1593/neo.111512 22355272 PMC3281940

[62] Gil Del Alcazar CR Hardebeck MC Mukherjee B Tomimatsu N Gao X Yan J. Inhibition of DNA double-strand break repair by the dual PI3K/mTOR inhibitor NVP-BEZ235 as a strategy for radiosensitization of glioblastoma. Clin Cancer Res. (2014) 20:1235–48. 10.1158/1078-0432.CCR-13-1607 24366691 PMC3947495

[63] Gupta S Ramjaun AR Haiko P Wang Y Warne PH Nicke B. Binding of ras to phosphoinositide 3-kinase p110alpha is required for ras-driven tumorigenesis in mice. Cell. (2007) 129:957–68. 10.1016/j.cell.2007.03.051 17540175

[64] Konstantinidou G Bey EA Rabellino A Schuster K Maira MS Gazdar AF. Dual phosphoinositide 3-kinase/mammalian target of rapamycin blockade is an effective radiosensitizing strategy for the treatment of non-small cell lung cancer harboring K-RAS mutations. Cancer Res. (2009) 69:7644–52. 10.1158/0008-5472.CAN-09-0823 19789349 PMC2760010

[65] Chen YH Wei MF Wang CW Lee HW Pan SL Gao M. Dual phosphoinositide 3-kinase/mammalian target of rapamycin inhibitor is an effective radiosensitizer for colorectal cancer. Cancer Lett. (2015) 357:582–90. 10.1016/j.canlet.2014.12.015 25497009

[66] Kuger S Graus D Brendtke R Gunther N Katzer A Lutyj P. Radiosensitization of glioblastoma cell lines by the dual PI3K and mTOR Inhibitor NVP-BEZ235 depends on drug-irradiation schedule. Transl Oncol. (2013) 6:169–79. 10.1593/tlo.12364 23544169 PMC3610553

[67] Potiron VA Abderrahmani R Giang E Chiavassa S Di Tomaso E Maira SM. Radiosensitization of prostate cancer cells by the dual PI3K/mTOR inhibitor BEZ235 under normoxic and hypoxic conditions. Radiother Oncol. (2013) 106:138–46. 10.1016/j.radonc.2012.11.014 23321494

[68] Zhu W Fu W Hu L. NVP-BEZ235, dual phosphatidylinositol 3-kinase/mammalian target of rapamycin inhibitor, prominently enhances radiosensitivity of prostate cancer cell line PC-3. Cancer Biother Radiopharm. (2013) 28:665–73. 10.1089/cbr.2012.1443 23768063

[69] Chang L Graham PH Hao J Ni J Bucci J Cozzi PJ. PI3K/Akt/mTOR pathway inhibitors enhance radiosensitivity in radioresistant prostate cancer cells through inducing apoptosis, reducing autophagy, suppressing NHEJ and HR repair pathways. Cell Death Dis. (2014) 5:e1437. 10.1038/cddis.2014.415 25275598 PMC4237243

[70] Schötz U Balzer V Brandt FW Frank Ziemann F Subtil FSB Rieckmann T. Dual PI3K/mTOR inhibitor NVP-BEZ235 enhances radiosensitivity of head and neck squamous cell carcinoma (HNSCC) cell lines due to suppressed double-strand break (DSB) repair by non-homologous end joining. Cancers. (2020) 12:467. 10.3390/cancers12020467 32085396 PMC7072694

[71] Jang NY Kim DH Cho BJ Choi EJ Lee JS Wu HG. Radiosensitization with combined use of olaparib and PI-103 in triple-negative breast cancer. BMC Cancer. (2015) 15:89. 10.1186/s12885-015-1090-7 25884663 PMC4355140

[72] Prevo R Deutsch E Sampson O Diplexcito J Cengel K Harper J. Class I PI3 kinase inhibition by the pyridinylfuranopyrimidine inhibitor PI-103 enhances tumor radiosensitivity. Cancer Res. (2008) 68:5915–23. 10.1158/0008-5472.CAN-08-0757 18632646

[73] Leiker AJ Degraff W Choudhuri R Sowers AL Thetford A Cook JA. Radiation enhancement of head and neck squamous cell carcinoma by the dual PI3K/mTOR inhibitor PF-05212384. Clin Cancer Res. (2015) 21:2792–801. 10.1158/1078-0432.CCR-14-3279 25724523 PMC4470749

[74] Tonlaar N Galoforo S Thibodeau BJ Ahmed S Wilson TG Yumpo Cardenas P. Antitumor activity of the dual PI3K/MTOR inhibitor, PF-04691502, in combination with radiation in head and neck cancer. Radiother Oncol. (2017) 124:504–12. 10.1016/j.radonc.2017.08.001 28823407

[75] Fokas E Im JH Hill S Yameen S Stratford M Beech J. Dual inhibition of the PI3K/mTOR pathway increases tumor radiosensitivity by normalizing tumor vasculature. Cancer Res. (2012) 72:239–48. 10.1158/0008-5472.CAN-11-2263 22108822

[76] Fokas E Yoshimura M Prevo R Higgins G Hackl W Maira SM. NVP-BEZ235 and NVP-BGT226, dual phosphatidylinositol 3-kinase/mammalian target of rapamycin inhibitors, enhance tumor and endothelial cell radiosensitivity. Radiat Oncol. (2012) 7:48. 10.1186/1748-717X-7-48 22452803 PMC3348043

[77] Holler M Grottke A Mueck K Manes J Jucker M Rodemann HP. Dual targeting of Akt and mTORC1 impairs repair of DNA double-strand breaks and increases radiation sensitivity of human tumor cells. PLoS ONE. (2016) 11:e0154745. 10.1371/journal.pone.0154745 27137757 PMC4854483

[78] Williams TM Flecha AR Keller P Ram A Karnak D Galban S. Cotargeting MAPK and PI3K signaling with concurrent radiotherapy as a strategy for the treatment of pancreatic cancer. Mol Cancer Ther. (2012) 11:1193–202. 10.1158/1535-7163.MCT-12-0098 22411900 PMC3349776

[79] Toulany M Iida M Keinath S Iyi FF Mueck K Fehrenbacher B. Dual targeting of PI3K and MEK enhances the radiation response of K-RAS mutated non-small cell lung cancer. Oncotarget. (2016) 7:43746–61. 10.18632/oncotarget.9670 27248324 PMC5190057

[80] Kuger S Flentje M Djuzenova CS. Simultaneous perturbation of the MAPK and the PI3K/mTOR pathways does not lead to increased radiosensitization. Radiat Oncol. (2015) 10:214. 10.1186/s13014-015-0514-5 26498922 PMC4619315

[81] Blas K Wilson TG Tonlaar N Galoforo S Hana A Marples B. Dual blockade of PI3K and MEK in combination with radiation in head and neck cancer. Clin Transl Radiat Oncol. (2018) 11:1–10. 10.1016/j.ctro.2018.04.003 30014041 PMC6019866

[82] Carrano AV. Chromosome aberrations and radiation-induced cell death II Predicted and observed cell survival. Mutat Res. (1973) 17:355–66. 10.1016/0027-5107(73)90007-9 4688369

[83] Dillon MT Good JS Harrington KJ. Selective targeting of the G2/M cell cycle checkpoint to improve the therapeutic index of radiotherapy. Clin Oncol. (2014) 26:257–65. 10.1016/j.clon.2014.01.009 24581946

[84] Moore PS Chang Y. Why do viruses cause cancer? Highlights of the first century of human tumour virology. Nat Rev Cancer. (2010) 10:878–89. 10.1038/nrc2961 21102637 PMC3718018

[85] Joerger AC Fersht AR. The p53 pathway: origins, inactivation in cancer, and emerging therapeutic approaches. Annu Rev Biochem. (2016) 85:375–404. 10.1146/annurev-biochem-060815-014710 27145840

[86] Qiu Z Oleinick NL Zhang J. ATR/CHK1 inhibitors and cancer therapy. Radiother Oncol. (2018) 126:450–64. 10.1016/j.radonc.2017.09.043 29054375 PMC5856582

[87] Zhang Y Hunter T. Roles of Chk1 in cell biology and cancer therapy. Int J Cancer. (2014) 134:1013–23. 10.1002/ijc.28226 23613359 PMC3852170

[88] Watanabe N Broome M Hunter T. Regulation of the human WEE1Hu CDK tyrosine 15-kinase during the cell cycle. EMBO J. (1995) 14:1878–91. 10.1002/j.1460-2075.1995.tb07180.x 7743995 PMC398287

[89] Krajewska M Heijink AM Bisselink YJ Seinstra RI Sillje HH De Vries EG. Forced activation of Cdk1 via wee1 inhibition impairs homologous recombination. Oncogene. (2013) 32:3001–8. 10.1038/onc.2012.296 22797065

[90] Godon C Cordelieres FP Biard D Giocanti N Megnin-Chanet F Hall J. PARP inhibition versus PARP-1 silencing: different outcomes in terms of single-strand break repair and radiation susceptibility. Nucleic Acids Res. (2008) 36:4454–64. 10.1093/nar/gkn403 18603595 PMC2490739

[91] Helleday T. The underlying mechanism for the PARP and BRCA synthetic lethality: clearing up the misunderstandings. Mol Oncol. (2011) 5:387–93. 10.1016/j.molonc.2011.07.001 21821475 PMC5528309

[92] Murai J Huang SY Das BB Renaud A Zhang Y Doroshow JH. Trapping of PARP1 and PARP2 by Clinical PARP Inhibitors. Cancer Res. (2012) 72:5588–99. 10.1158/0008-5472.CAN-12-2753 23118055 PMC3528345

[93] Iliakis G. Backup pathways of NHEJ in cells of higher eukaryotes: cell cycle dependence. Radiother Oncol. (2009) 92:310–5. 10.1016/j.radonc.2009.06.024 19604590

[94] Bryant HE Schultz N Thomas HD Parker KM Flower D Lopez E. Specific killing of BRCA2-deficient tumours with inhibitors of poly(ADP-ribose) polymerase. Nature. (2005) 434:913–7. 10.1038/nature03443 15829966

[95] Busch CJ Kroger MS Jensen J Kriegs M Gatzemeier F Petersen C. G2-checkpoint targeting and radiosensitization of HPV/p16-positive HNSCC cells through the inhibition of Chk1 and Wee1. Radiother Oncol. (2017) 122:260–6. 10.1016/j.radonc.2016.11.017 27939202

[96] Vance S Liu E Zhao L Parsels JD Parsels LA Brown JL. Selective radiosensitization of p53 mutant pancreatic cancer cells by combined inhibition of Chk1 and PARP1. Cell Cycle. (2011) 10:4321–9. 10.4161/cc.10.24.18661 22134241 PMC3272262

[97] Guster JD Weissleder SV Busch CJ Kriegs M Petersen C Knecht R. The inhibition of PARP but not EGFR results in the radiosensitization of HPV/p16-positive HNSCC cell lines. Radiother Oncol. (2014) 113:345–51. 10.1016/j.radonc.2014.10.011 25467050

[98] Karnak D Engelke CG Parsels LA Kausar T Wei D Robertson JR. Combined inhibition of Wee1 and PARP1/2 for radiosensitization in pancreatic cancer. Clin Cancer Res. (2014) 20:5085–96. 10.1158/1078-0432.CCR-14-1038 25117293 PMC4184968

[99] Cuneo KC Morgan MA Davis MA Parcels LA Parcels J Karnak D. Wee1 kinase inhibitor AZD1775 radiosensitizes hepatocellular carcinoma regardless of TP53 mutational status through induction of replication stress. Int J Radiat Oncol Biol Phys. (2016) 95:782–90. 10.1016/j.ijrobp.2016.01.028 26975930 PMC6644066

[100] Parsels LA Karnak D Parsels JD Zhang Q Velez-Padilla J Reichert ZR. PARP1 trapping and DNA replication stress enhance radiosensitization with combined WEE1 and PARP inhibitors. Mol Cancer Res. (2018) 16:222–32. 10.1158/1541-7786.MCR-17-0455 29133592 PMC5805596

[101] Molkentine JM Molkentine DP Bridges KA Xie T Yang L Sheth A. Targeting DNA damage response in head and neck cancers through abrogation of cell cycle checkpoints. Int J Radiat Biol. 25:1–8. 10.1080/09553002.2020.1730014 32073931 PMC7483862

[102] Cimprich KA Cortez D. ATR: an essential regulator of genome integrity. Nat Rev Mol Cell Biol. (2008) 9:616–27. 10.1038/nrm2450 18594563 PMC2663384

[103] Fokas E Prevo R Hammond EM Brunner TB Mckenna WG Muschel RJ. Targeting ATR in DNA damage response and cancer therapeutics. Cancer Treat Rev. (2014) 40:109–17. 10.1016/j.ctrv.2013.03.002 23583268

[104] Carruthers RD Ahmed SU Ramachandran S Strathdee K Kurian KM Hedley A. Replication stress drives constitutive activation of the DNA damage response and radioresistance in glioblastoma stem-like cells. Cancer Res. (2018) 78:5060–71. 10.1158/0008-5472.CAN-18-0569 29976574 PMC6128404

[105] Ahmed SU Carruthers R Gilmour L Yildirim S Watts C Chalmers AJ. Selective inhibition of parallel DNA damage response pathways optimizes radiosensitization of glioblastoma stem-like cells. Cancer Res. (2015) 75:4416–28. 10.1158/0008-5472.CAN-14-3790 26282173

[106] Wera AC Lobbens A Stoyanov M Lucas S Michiels C. Radiation-induced synthetic lethality: combination of poly(ADP-ribose) polymerase and RAD51 inhibitors to sensitize cells to proton irradiation. Cell Cycle. (2019) 18:1770–83. 10.1080/15384101.2019.1632640 31238782 PMC6649553

[107] Zeng L Beggs RR Cooper TS Weaver AN Yang ES. Combining Chk1/2 inhibition with cetuximab and radiation enhances *in vitro* and *in vivo* cytotoxicity in head and neck squamous cell carcinoma. Mol Cancer Ther. (2017) 16:591–600. 10.1158/1535-7163.MCT-16-0352 28138028 PMC5560482

[108] Mahaney BL Meek K Lees-Miller SP. Repair of ionizing radiation-induced DNA double-strand breaks by non-homologous end-joining. Biochem J. (2009) 417:639–50. 10.1042/BJ20080413 19133841 PMC2975036

[109] Goodwin JF Knudsen KE. Beyond DNA repair: DNA-PK function in cancer. Cancer Discov. (2014) 4:1126–39. 10.1158/2159-8290.CD-14-0358 25168287 PMC4184981

[110] Hafsi H Dillon MT Barker HE Kyula JN Schick U Paget JT. Combined ATR and DNA-PK inhibition radiosensitizes tumor cells independently of their p53 status. Front Oncol. (2018) 8:245. 10.3389/fonc.2018.00245 30057890 PMC6053502

[111] Blandin AF Renner G Lehmann M Lelong-Rebel I Martin S Dontenwill M. beta1 integrins as therapeutic targets to disrupt hallmarks of cancer. Front Pharmacol. (2015) 6:279. 10.3389/fphar.2015.00279 26635609 PMC4656837

[112] Eke I Cordes N. Focal adhesion signaling and therapy resistance in cancer. Semin Cancer Biol. (2015) 31:65–75. 10.1016/j.semcancer.2014.07.009 25117005

[113] Eke I Zscheppang K Dickreuter E Hickmann L Mazzeo E Unger K Simultaneous beta1 integrin-EGFR targeting and radiosensitization of human head and neck cancer. J Natl Cancer Inst. (2015) 107 10.1093/jnci/dju419 25663685

[114] Eke I Cordes N. Dual targeting of EGFR and focal adhesion kinase in 3D grown HNSCC cell cultures. Radiother Oncol. (2011) 99:279–86. 10.1016/j.radonc.2011.06.006 21704406

[115] Zscheppang K Kurth I Wachtel N Dubrovska A Kunz-Schughart LA Cordes N. Efficacy of beta1 integrin and EGFR targeting in sphere-forming human head and neck cancer cells. J Cancer. (2016) 7:736–45. 10.7150/jca.14232 27076856 PMC4829561

[116] Poschau M Dickreuter E Singh-Muller J Zscheppang K Eke I Liersch T. EGFR and beta1-integrin targeting differentially affect colorectal carcinoma cell radiosensitivity and invasion. Radiother Oncol. (2015) 116:510–6. 10.1016/j.radonc.2015.06.005 26096850

[117] Klapproth E Dickreuter E Zakrzewski F Seifert M Petzold A Dahl A. Whole exome sequencing identifies mTOR and KEAP1 as potential targets for radiosensitization of HNSCC cells refractory to EGFR and beta1 integrin inhibition. Oncotarget. (2018) 9:18099–114. 10.18632/oncotarget.24266 29719593 PMC5915060

[118] Koppenhagen P Dickreuter E Cordes N. Head and neck cancer cell radiosensitization upon dual targeting of c-Abl and beta1-integrin. Radiother Oncol. (2017) 124:370–8. 10.1016/j.radonc.2017.05.011 28578803

[119] Vehlow A Klapproth E Storch K Dickreuter E Seifert M Dietrich A. Adhesion- and stress-related adaptation of glioma radiochemoresistance is circumvented by beta1 integrin/JNK co-targeting. Oncotarget. (2017) 8:49224–37. 10.18632/oncotarget.17480 28514757 PMC5564763

[120] Morimoto RI. Cells in stress: transcriptional activation of heat shock genes. Science. (1993) 259:1409–10. 10.1126/science.8451637 8451637

[121] Jego G Hazoume A Seigneuric R Garrido C. Targeting heat shock proteins in cancer. Cancer Lett. (2013) 332:275–85. 10.1016/j.canlet.2010.10.014 21078542

[122] Schopf FH Biebl MM Buchner J. The HSP90 chaperone machinery. Nat Rev Mol Cell Biol. (2017) 18:345–60. 10.1038/nrm.2017.20 28429788

[123] Dai C Sampson SB. HSF1: guardian of proteostasis in cancer. Trends Cell Biol. (2016) 26:17–28. 10.1016/j.tcb.2015.10.011 26597576 PMC4722819

[124] Alexandrova EM Marchenko ND. Mutant p53 - heat shock response oncogenic cooperation: a new mechanism of cancer cell survival. Front Endocrinol. (2015) 6:53. 10.3389/fendo.2015.00053 25954247 PMC4406088

[125] Dote H Burgan WE Camphausen K Tofilon PJ. Inhibition of hsp90 compromises the DNA damage response to radiation. Cancer Res. (2006) 66:9211–20. 10.1158/0008-5472.CAN-06-2181 16982765

[126] Elaimy AL Ahsan A Marsh K Pratt WB Ray D Lawrence TS. ATM is the primary kinase responsible for phosphorylation of Hsp90alpha after ionizing radiation. Oncotarget. (2016) 7:82450–7. 10.18632/oncotarget.12557 27738310 PMC5347704

[127] Schilling D Garrido C Combs SE Multhoff G. The Hsp70 inhibiting peptide aptamer A17 potentiates radiosensitization of tumor cells by Hsp90 inhibition. Cancer Lett. (2017) 390:146–52. 10.1016/j.canlet.2017.01.015 28108313

[128] Schilling D Kuhnel A Konrad S Tetzlaff F Bayer C Yaglom J. Sensitizing tumor cells to radiation by targeting the heat shock response. Cancer Lett. (2015) 360:294–301. 10.1016/j.canlet.2015.02.033 25721082

[129] Stuhmer T Iskandarov K Gao Z Bumm T Grella E Jensen MR. Preclinical activity of the novel orally bioavailable HSP90 inhibitor NVP-HSP990 against multiple myeloma cells. Anticancer Res. (2012) 32:453–62. 22287732

[130] Djuzenova CS Fiedler V Katzer A Michel K Deckert S Zimmermann H Dual PI3K- and mTOR-inhibitor PI-103 can either enhance or reduce the radiosensitizing effect of the Hsp90 inhibitor NVP-AUY922 in tumor cells: the role of drug-irradiation schedule. Oncotarget. (2016) 7:38191–209. 10.18632/oncotarget.9501 27224913 PMC5122382

[131] Wachsberger PR Lawrence YR Liu Y Rice B Feo N Leiby B. Hsp90 inhibition enhances PI-3 kinase inhibition and radiosensitivity in glioblastoma. J Cancer Res Clin Oncol. (2014) 140:573–82. 10.1007/s00432-014-1594-6 24500492 PMC11823890

[132] Dungey FA Caldecott KW Chalmers AJ. Enhanced radiosensitization of human glioma cells by combining inhibition of poly(ADP-ribose) polymerase with inhibition of heat shock protein 90. Mol Cancer Ther. (2009) 8:2243–54. 10.1158/1535-7163.MCT-09-0201 19671736 PMC2728724

[133] Tao Z Le Blanc JM Wang C Zhan T Zhuang H Wang P. Coadministration of trametinib and palbociclib radiosensitizes KRAS-mutant non-small cell lung cancers *in vitro* and *in vivo*. Clin Cancer Res. (2016) 22:122–33. 10.1158/1078-0432.CCR-15-0589 26728409

[134] Groselj B Sharma NL Hamdy FC Kerr M Kiltie AE Histone deacetylase inhibitors as radiosensitisers: effects on DNA damage signalling and repair. Br J Cancer. (2013) 108:748–54. 10.1038/bjc.2013.21 23361058 PMC3590661

[135] Moertl S Payer S Kell R Winkler K Anastasov N Atkinson MJ. Comparison of radiosensitization by HDAC inhibitors CUDC-101 and SAHA in pancreatic cancer cells. Int J Mol Sci. (2019) 20:3259. 10.3390/ijms20133259 31269745 PMC6651299

[136] Loriot Y Mordant P Dugue D Geneste O Gombos A Opolon P. Radiosensitization by a novel Bcl-2 and Bcl-XL inhibitor S44563 in small-cell lung cancer. Cell Death Dis. (2014) 5:e1423. 10.1038/cddis.2014.365 25232677 PMC4540189

[137] Xiao R An Y Ye W Derakhshan A Cheng H Yang X. Dual Antagonist of cIAP/XIAP ASTX660 Sensitizes HPV(-) and HPV(+) Head and neck cancers to TNFalpha, TRAIL, and radiation therapy. Clin Cancer Res. (2019) 25:6463–74. 10.1158/1078-0432.CCR-18-3802 31266830 PMC6825532

[138] Argiris A Bauman JE Ohr J Gooding WE Heron DE Duvvuri U Phase II randomized trial of radiation therapy, cetuximab, and pemetrexed with or without bevacizumab in patients with locally advanced head and neck cancer. Ann Oncol. (2016) 27:1594–600. 10.1093/annonc/mdw204 27177865 PMC6279075

[139] Bendell JC Meluch A Peyton J Rubin M Waterhouse D Webb C. A phase II trial of preoperative concurrent chemotherapy/radiation therapy plus bevacizumab/erlotinib in the treatment of localized esophageal cancer. Clin Adv Hematol Oncol. (2012) 10:430–7. 22895283

[140] Socinski MA Stinchcombe TE Moore DT Gettinger SN Decker RH Petty WJ. Incorporating bevacizumab and erlotinib in the combined-modality treatment of stage III non-small-cell lung cancer: results of a phase I/II trial. J Clin Oncol. (2012) 30:3953–9. 10.1200/JCO.2012.41.9820 23045594

[141] Lee EQ Kaley TJ Duda DG Schiff D Lassman AB Wong ET A multicenter, phase II, randomized, noncomparative clinical trial of radiation and temozolomide with or without vandetanib in newly diagnosed glioblastoma patients. Clin Cancer Res. (2015) 21:3610–8. 10.1158/1078-0432.CCR-14-3220 25910950 PMC4790106

[142] Blaszkowsky LS Ryan DP Szymonifka J Borger DR Zhu AX Clark JW. Phase I/II study of neoadjuvant bevacizumab, erlotinib and 5-fluorouracil with concurrent external beam radiation therapy in locally advanced rectal cancer. Ann Oncol. (2014) 25:121–6. 10.1093/annonc/mdt516 24356623 PMC4271130

[143] Das P Eng C Rodriguez-Bigas MA Chang GJ Skibber JM You YN. Preoperative radiation therapy with concurrent capecitabine, bevacizumab, and erlotinib for rectal cancer: a phase 1 trial. Int J Radiat Oncol Biol Phys. (2014) 88:301–5. 10.1016/j.ijrobp.2013.10.034 24315563 PMC5592790

[144] Harrington K Berrier A Robinson M Remenar E Housset M De Mendoza FH. Randomised Phase II study of oral lapatinib combined with chemoradiotherapy in patients with advanced squamous cell carcinoma of the head and neck: rationale for future randomised trials in human papilloma virus-negative disease. Eur J Cancer. (2013) 49:1609–18. 10.1016/j.ejca.2012.11.023 23265705

[145] Harrington K Temam S Mehanna H D'cruz A Jain M D'onofrio I. Postoperative adjuvant lapatinib and concurrent chemoradiotherapy followed by maintenance lapatinib monotherapy in high-risk patients with resected squamous cell carcinoma of the head and neck: a phase III, randomized, double-blind, placebo-controlled study. J Clin Oncol. (2015) 33:4202–9. 10.1200/JCO.2015.61.4370 26527790

[146] Chen AY Okunieff P Pommier Y Mitchell JB. Mammalian DNA topoisomerase I mediates the enhancement of radiation cytotoxicity by camptothecin derivatives. Cancer Res. (1997) 57:1529–36. 9108456

[147] Bernhard EJ Mckenna WG Hamilton AD Sebti SM Qian Y Wu JM. Inhibiting Ras prenylation increases the radiosensitivity of human tumor cell lines with activating mutations of ras oncogenes. Cancer Res. (1998) 58:1754–61. 9563495

[148] Sartor CI. Biological modifiers as potential radiosensitizers: targeting the epidermal growth factor receptor family. Semin Oncol. (2000) 27, 15–20; discussion 92–100. 10.1016/B978-0-12-398342-8.00007-0 11236022

[149] Harari PM Huang SM. Epidermal growth factor receptor modulation of radiation response: preclinical and clinical development. Semin Radiat Oncol. (2002) 12:21–6. 10.1053/srao.2002.34865 12174341

[150] Rieckmann T Kriegs M. The failure of cetuximab-based de-intensified regimes for HPV-positive OPSCC: a radiobiologists perspective. Clin Transl Radiat Oncol. (2019) 17:47–50. 10.1016/j.ctro.2019.05.003 31206086 PMC6558227

[151] Tang L Wei F Wu Y He Y Shi L Xiong F. Role of metabolism in cancer cell radioresistance and radiosensitization methods. J Exp Clin Cancer Res. (2018) 37:87. 10.1186/s13046-018-0758-7 29688867 PMC5914062

[152] Lewis JE Singh N Holmila RJ Sumer BD Williams NS Furdui CM. Targeting NAD(+) metabolism to enhance radiation therapy responses. Semin Radiat Oncol. (2019) 29:6–15. 10.1016/j.semradonc.2018.10.009 30573185 PMC6310039

[153] Peitzsch C Kurth I Ebert N Dubrovska A Baumann M. Cancer stem cells in radiation response: current views and future perspectives in radiation oncology. Int J Radiat Biol. (2019) 95:900–11. 10.1080/09553002.2019.1589023 30897014

[154] Shibata M Hoque MO. Targeting cancer stem cells: a strategy for effective eradication of cancer. Cancers. (2019) 11:732. 10.3390/cancers11050732 31137841 PMC6562442

[155] Konings K Vandevoorde C Baselet B Baatout S Moreels M Combination therapy with charged particles and molecular targeting: a promising avenue to overcome radioresistance. Front Oncol. (2020) 10:128 10.3389/fonc.2020.00128 32117774 PMC7033551

[156] Kriegs M Clauditz TS Hoffer K Bartels J Buhs S Gerull H. Analyzing expression and phosphorylation of the EGF receptor in HNSCC. Sci Rep. (2019) 9:13564. 10.1038/s41598-019-49885-5 31537844 PMC6753061

[157] Lord CJ Ashworth A. PARP inhibitors: synthetic lethality in the clinic. Science. (2017) 355:1152–8. 10.1126/science.aam7344 28302823 PMC6175050

[158] Sorensen CS Hansen LT Dziegielewski J Syljuasen RG Lundin C Bartek J. The cell-cycle checkpoint kinase Chk1 is required for mammalian homologous recombination repair. Nat Cell Biol. (2005) 7:195–201. 10.1038/ncb1212 15665856

[159] Buisson R Niraj J Rodrigue A Ho CK Kreuzer J Foo TK. Coupling of homologous recombination and the checkpoint by ATR. Mol Cell. (2017) 65:336–46. 10.1016/j.molcel.2016.12.007 28089683 PMC5496772

[160] Kim D Liu Y Oberly S Freire R Smolka MB. ATR-mediated proteome remodeling is a major determinant of homologous recombination capacity in cancer cells. Nucleic Acids Res. (2018) 46:8311–25. 10.1093/nar/gky625 30010936 PMC6144784

[161] Huang J Meng L Yang B Sun S Luo Z Chen H. Safety profile of epidermal growth factor receptor tyrosine kinase inhibitors: a disproportionality analysis of FDA adverse event reporting system. Sci Rep. (2020) 10:4803. 10.1038/s41598-020-61571-5 32179761 PMC7075865

[162] Bonomo P Loi M Desideri I Olmetto E Delli Paoli C Terziani F. Incidence of skin toxicity in squamous cell carcinoma of the head and neck treated with radiotherapy and cetuximab: a systematic review. Crit Rev Oncol Hematol. (2017) 120:98–110. 10.1016/j.critrevonc.2017.10.011 29198343

[163] Crane CH Eng C Feig BW Das P Skibber JM Chang GJ. Phase II trial of neoadjuvant bevacizumab, capecitabine, and radiotherapy for locally advanced rectal cancer. Int J Radiat Oncol Biol Phys. (2010) 76:824–30. 10.1016/j.ijrobp.2009.02.037 19464823

[164] Niyazi M Ganswindt U Schwarz SB Kreth FW Tonn JC Geisler J. Irradiation and bevacizumab in high-grade glioma retreatment settings. Int J Radiat Oncol Biol Phys. (2012) 82:67–76. 10.1016/j.ijrobp.2010.09.002 21030162

[165] Spigel DR Hainsworth JD Yardley DA Raefsky E Patton J Peacock N. Tracheoesophageal fistula formation in patients with lung cancer treated with chemoradiation and bevacizumab. J Clin Oncol. (2010) 28:43–8. 10.1200/JCO.2009.24.7353 19901100

[166] Wehler T Thomas M Schumann C Bosch-Barrera J Vinolas Segarra N Dickgreber NJ. A randomized, phase 2 evaluation of the CHK1 inhibitor, LY2603618, administered in combination with pemetrexed and cisplatin in patients with advanced nonsquamous non-small cell lung cancer. Lung Cancer. (2017) 108:212–6. 10.1016/j.lungcan.2017.03.001 28625637

[167] Sausville E Lorusso P Carducci M Carter J Quinn MF Malburg L. Phase I dose-escalation study of AZD7762, a checkpoint kinase inhibitor, in combination with gemcitabine in US patients with advanced solid tumors. Cancer Chemother Pharmacol. (2014) 73:539–49. 10.1007/s00280-014-2380-5 24448638 PMC4486055

