# Supplementary material for: Improving the Efficacy of Tumor Radiosensitization Through Combined Molecular Targeting
Source: Front Oncol. 2020 Aug 4;10:1260. doi: 10.3389/fonc.2020.01260 (PMC7438822; doi:10.3389/fonc.2020.01260)
Supplement: Supplementary file 1 [file Image_1.pdf]

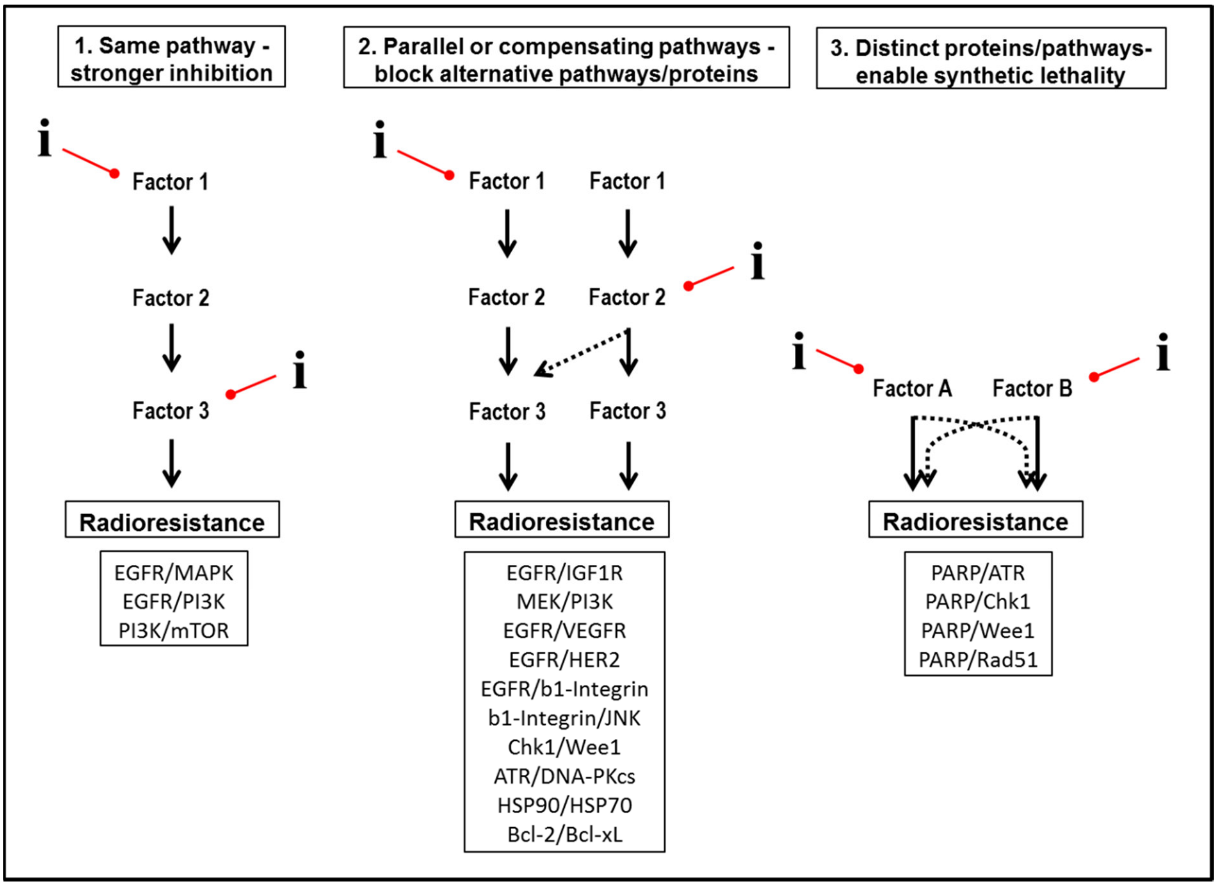

Supplementary figure 1. Basic strategies for dual molecular targeting and examples of pairs of targeted proteins. Note that inhibition can take place at various points in the pathways and that it may not always be possible to clearly distinguish between strategies 2 and 3. i=inhibitor.
